# Synthesis and Computational Characterization of Organic UV-Dyes for Cosensitization of Transparent Dye-Sensitized Solar Cells

**DOI:** 10.3390/molecules26237336

**Published:** 2021-12-03

**Authors:** Rua B. Alnoman, Eman Nabil, Shazia Parveen, Mohamed Hagar, Mohamed Zakaria, Ahmed A. Hasanein

**Affiliations:** 1 Department of Chemistry, Faculty of Science, Taibah University, Yanbu 4642, Saudi Arabia; rua-b-n@live.co.uk (R.B.A.); shazia021@gmail.com (S.P.); 2 Department of Chemistry, Faculty of Science, Alexandria University, Alexandria 21321, Egypt; m.zak_8770@hotmail.com (M.Z.); Ahemedhasanein@Alexu.edu.eg (A.A.H.)

**Keywords:** dye-sensitized solar cells, computational characterization, organic UV-dyes, cosensitization

## Abstract

The fabrication of colorless and see-through dye-sensitized solar cells (DSCs) requires the photosensitizers to have little or no absorption in the visible light region of the solar spectrum. However, a trade-off between transparency and power conversion efficiency (PCE) has to be tackled, since most transparent DSCs are showing low PCE when compared to colorful and opaque DSCs. One strategy to increase PCE is applying two cosensitizers with selective conversion of the UV and NIR radiation, therefore, the non-visible part only is absorbed. In this study, we report synthesis of novel five UV-selective absorbers, based on diimide and Schiff bases incorporating carboxyl and pyridyl anchoring groups. A systematic computational investigation using density functional theory (DFT) and time-dependent DFT approaches was employed to evaluate their prospect of application in transparent DSCs. Experimental UV/Vis absorption spectra showed that all dyes exhibit an absorption band covering the mid/near-UV region of solar spectrum, with a bathochromic shift and a hyperchromic shifts for Py-1 dye. Computational results showed that the studied dyes satisfied the basic photophysical and energetics requirements of operating DSC as well as the stability and thermodynamical spontaneity of adsorption onto surface of TiO_2_. However, results revealed outperformance of the thienothiophene core-containing Py-1 UV-dye, owing to its advantageous structural attributes, improved conjugation, intense emission, large Stokes shift and maximum charge transferred to the anchor. Chemical compatibility of Py-1 dye was then theoretically investigated as a potential cosensitizer of a reference VG20-C2 NIR-dye. By the judicious selection of pyridyl anchor-based UV-absorber (Py-1) and carboxyl anchor-based NIR-absorber (VG20), the advantage of the optical complementarity and selectivity of different TiO_2_-adsorption-site (Lewis- and Bronsted-acidic) can be achieved. An improved overall PCE is estimated accordingly.

## 1. Introduction

One of the emerging environmental technologies is building integrated (BI) solar-powered windows, with built-in solar panels, for converting sunlight into electricity [1]. Dye-sensitized solar cell (DSC) is one of the eco-friendly sources of solar energy that has a great potential to be applied in solar windows owing to its high average visible transmittance (AVT) and ability to operate in poor or ambient sunlight conditions [2,3,4]. Inspired by the crucial role of chlorophyll in the photosynthesis process, O’Regan and Grätzel reported first DSC device using fabrication process called “sensitization” of mesoporous titanium oxide (TiO_2_) film using photosensitizer (dye) [5]. In DSCs, the sensitizer that adsorbs chemically to the semiconductor is the accountable component of capturing and converting light energy. The photoelectron transport in such solar cells can be divided into four steps [6]: (1) excitation of dye molecules through light absorption; (2) subsequently injection of photo-excited electrons into the conduction band (*CB*) of the semiconductor (frequently TiO_2_); (3) oxidized dye molecules obtain electrons from redox couple: (I^−^/I_3_^−^ or Co^3+^/Co^2+^) so that regeneration of dye is achievable; (4) reduction of the oxidized redox mediator at the counter electrode to complete the circuit. Yet, during the operating cycle of DSCs, there are unwanted competing loss pathways leading to lower overall power conversion efficiency (PCE) of the device. Namely, decay of the excited dye to the ground state and recombination of the injected electrons in the *CB* of semiconductor with the adsorbed oxidized dye molecules or with ions in the electrolyte solution [7].

Cosensitization of the semiconducting electrode, with two or more photosensitizers with complementary absorption spectra, is the direct solution to overcome the partial absorption coverage of solar emission spectrum resulted from using single sensitizer [8]. For achieving a panchromatic absorption spectrum in traditional opaque cosensitized DSC (co-DSC) systems, opaque absorbers (cosensitizers) are required to cover the entire visible region and extend toward the near-infrared (NIR) region of solar spectrum.

In contrast, transparent and colorless co-DSCs/DSCs devices require a selective conversion of the UV and/or NIR regions of the solar spectrum through which only the nonvisible part of the solar spectrum is converted into electricity [9]. Since the human eye typically responds to visible light from 400 to 700 nm, cosensitizers should have little or no absorption in the visible light region with high human eye sensitivity (500–600 nm). In DSCs, tailoring the coloration and level of transparency can be achieved by modulating the absorption range of photosensitizers and the electrode thickness. Thickness of electrode can be controlled by employing sensitizers with high molar extinction coefficients which permit the fabrication of DSCs with thinner TiO_2_ films [10]. In solar-powered windows, TiO_2_ layer is supposed to be sufficiently thin in order to not be seen. Indeed, previous work in organic and organometallic DSCs has shown ongoing progress in the development of transparent solar cells. A see-through DSC, with over 10% efficiency, was proposed by Wang et al., who fabricated a semitransparent cell using donor–acceptor thienochrysenocarbazole-based organic dyes with experimental absorption maximum (λ_max_) of 562 to 582 nm in toluene solvent [11]. Similarly, Yuan et al. employed donor–acceptor organic dyes for fabrication of goldenrod, crimson, red, and sapphire blue semitransparent cells with power conversion efficiencies of 3.5%, 8.2%, 7.6%, and 10.1%, respectively [12]. Light-driven adjustable optical transmission due to photochromic sensitizers was proposed by Demadrille et al., who achieved a maximum PCE of up to 4.17% while exhibiting a reversible change of color under irradiation [13]. Earlier, Han et al. reported a semitransparent DSC based on a UV/NIR cosensitization system. The green-colored see-through DSC was fabricated with Y1 and HSQ5 cosensitizers, taking the advantage of the dyes’ strong absorptions in 400 and 700 nm, respectively, which are low-eye-sensitivity regions [14]. The most remarkable achievement was recently reported by Sauvage et al., who were able to fabricate fully transparent and colorless DSCs by considering the human eye photopic response to optimize NIR absorbing dyes [15]. The reported UV-Vis spectra of theses NIR-selective dyes have strong and sharp absorption peak beyond 800 nm due to S0–S1 electronic transition. Dyes are based on symmetrical polymethine cyanine, coded VG20-Cx, with x values varying from 2 to 16 carbons on the alkyl chain, and the authors reported PCE of 3.1% and a maximum of 76% average visible transmittance. Diimides, which are part of a larger class of molecules known as rylenes dye, come in symmetrical and asymmetrical forms, depending on how they’re made [16]. Tunable features of diimides-based derivatives include quantum yields of near-unity fluorescence [17], and strong electron-accepting properties enable them to be employed as industrial colorants, both as dyes (soluble) and pigments (insoluble) [18], for the manufacturing of soft electrical devices [19] and organic electronics [20]. The imine or azomethine (–C=N–) functional group is found in Schiff bases. Hugo Schiff [21,22] described these as the condensation products of primary amines with carbonyl compounds for the first time. Schiff bases are a type of organic chemicals that has a wide range of uses in a variety of domains, including analytical, biological, and inorganic chemistry [23]. In this context, we report synthesis of five new symmetrical UV-selective organic dyes based on diimide (CA-1) and azomethine (CA-2, Py-1, Py-2 and Py-3). Subsequently, quantum chemical computations were performed to investigate their potential application as photosensitizers in DSCs and as promising cosensitizers of the NIR-selective VG20-C_2_ dye, reported by Sauvage et al. [15]. First, density functional theory (DFT) combined with time-dependent density functional theory (TD-DFT) calculations, were performed to qualitatively (or even quantitatively) examine the electrochemical and photophysical properties of the five new organic dyes matching the requirements for efficient DSCs. Secondly, the promising dye is shortlisted for testing as cosensitizer with the VG20-C_2_ dye. For efficient cosensitization, additional requirements are to be considered, namely chemical compatibility between cosensitizers and complementarity of their absorption spectra. This screen suggests the judicious choice of the UV-selective Py-1 dye as a very promising cosensitizer of the NIR-selective VG20-C_2_ dye. Such a strategy of cosensitization between UV and NIR dyes, aims for selectively harvesting light in the invisible region while transmitting light in the visible region. Hence, it should provide a rational approach to enhance overall PCE, and in the meantime retain fully transparency of DSCs for photovoltaic (PV) windows applications. 

## 2. Methodology

### 2.1. Experimental Methods and Materials

**Scheme 1:**
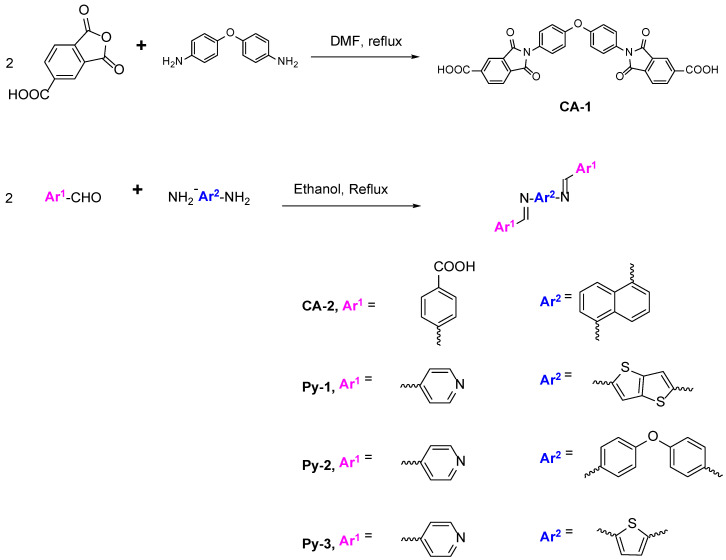
Synthesis of diimide CA-1 and Schiff bases CA-2 and Py-1-3.

The compounds under investigation have been synthesized according to the following Scheme 1:

#### 2.1.1. General Procedure of the Synthesis of Diimide CA-1

In DMF (5 mL), a mixture of 1,2,4-benzenetricarboxylic anhydride (0.34 mmol) and aromatic diamine (0.17 mmol) was refluxed for 6–8 h according to the TLC (hexane-ethyl acetate, 1:10). The reaction mixture was allowed to cool; then filtrated to collect the precipitate, which was subsequently recrystallized from DMF

#### 2.1.2. General Procedure of the Synthesis of Schiff Bases CA-2 and Py-1-3

Equimolar amount of aryldialdehyde (4.1 mmol) and arylamine (8.2 mmol) in ethanol (10 mL) were refluxed for two hours. The reaction mixture was allowed to cool and the separated product filtered. The obtained solid was recrystallized from ethanol.

*2,2′-[Oxybis(1,4-phenylene)]bis(1H-isoindole-1,3(2H)-dione-5-carboxylic acid)*, (**CA-1**). Yield 75%, m.p. > 300 °C, FTIR (KBr, cm^−1^): 3525–2810 (m, br, COOH), 1780 (C=O), 1730 (C=O), 1608 (C=C). ^1^HNMR (500 MHz, DMSO-*d*_6_), δ: 10.01 (s, 2H, OH), 8.65 (s, 1H), 8.43 (s, 1H), 7.98 (d, *J* = 8.5 Hz 4H), 7.70 (d, *J* = 8.5 Hz 4H), 7.58–7.47 (m, 2H), 7.40–7.20 (m, 2H), ^13^C NMR (175 MHz, CDCl_3_), δ: 167.37, 158, 156.46, 148, 141, 134.47, 131.79, 128.21, 127.07, 123.81, 119.56.

*Diphenylidene-4,4′-dicarboxy-naphthalene-1,5-diamine*, (**CA-2**). Yield 90%, m.p. = 251 °C, FTIR (KBr, cm^−1^): 3500–2820 (m, br, COOH), 1701 (C=O), 1626 (CH=N), 1580 (C=C). ^1^HNMR (500 MHz, DMSO-*d*_6_), δ: 10.49 (s, 2H, OH), 8.24 (s, 2H, CH=N), 8.31 (d, *J* = 8.6 Hz, 4H), 7.87 (d, *J* = 8.4 Hz, 4H), 7.48–7.35 (m, 6H).

*4-(pyridin-4-ylimino)methyl)thieno [3,2-b]thiophen-5-yl)methylene)pyridin-4-amine*, (**Py-1**). Yield 85%, m.p. > 300 °C, FTIR (KBr, cm^−1^): 3010, 1618 (C=N). 1H NMR (500 MHz, CDCl_3_): δ 8.29 (s, 2H, CH=N), 7.78 (d, *J* = 8.4 Hz, 4H, Py), 7.14 (d, *J* = 8.4 Hz, 4H, Py), 6.98 (s, 2H, thioph).

*4-((pyridin-4-yl)methyleneamino)phenoxy)-N-((pyridin-4-yl)methylene)benzenamine*, (**Py-2**). Yield 82%, m.p. = 285 °C (decomp.), FTIR (KBr, cm^−1^): 1626 (CH=N), 1580 (C=C), ^1^H NMR (500 MHz, CDCl_3_) δ: 9.01 (s, 2H, CH=N), 8.71 (d, *J* = 8.5 Hz, 4H), 8.45 (d, *J* = 8.5 Hz, 4H), 8.39–8.56 (m, 4H), 7.50–6.86 (m, 4H).

*4-(pyridin-4-ylimino)methyl)thiophen-2-yl)methylene)pyridin-4-amine*, (**Py-3**). Yield 88%, m.p. = 280 °C, FTIR (KBr, cm^−1^): 3018, 1636 (C=N). 1H NMR (500 MHz, CDCl_3_): δ 8.60 (s, 2H, CH=N), 8.04 (d, *J* = 8.5 Hz, 4H, Py), 7.49 (s, 2H, thioph), 7.35 (d, *J* = 8.5 Hz, 4H, Py). ^13^C NMR, δ 156, 146, 144, 132, 131, 129.

#### 2.1.3. Optical Spectroscopy

The UV/Vis absorption spectra of solution-based free dyes (in *N*,*N*-dimethylformamide) were determined using a Thermo Scientific Evolution 201/220 UV-Visible Spectrophotometers (Mettler-Toledo India Private Limited, Mumbai, India). The uncertainty in the UV/vis measurement is 2 nm.

### 2.2. Computational Methodology

#### 2.2.1. Ground State Studies of Isolated Dyes

The ground state geometries of all the new synthesized dyes as well as the reference dye (VG20-C_2_) were optimized using DFT method with the M06-2X/6-311g(d,p) level of theory [24,25]. This method is widely accepted and reliable in probing the non-covalent interactions (NCIs) [26] which will be assessed for the electronic structure of the studied dimer. The effect of N,N-dimethylformamide (DMF), the solvent that was used in measuring UV-Vis spectra of new synthesized dyes, was accounted for using the self-consistent reaction field (SCRF) based on the conductor-like polarizable continuum model (C-PCM). Frequency calculations at the same level of theory were subsequently performed in order to verify that the optimized geometrical structures were converged to true stationary points not saddle points. All geometries were optimized with tight convergence criteria corresponding to Max force = 1.5 × 10^−5^ and RMS force = 1.0 × 10^−5^ Hartree Bohr^−1^ as well as Max displacement = 6.0 × 10^−5^ and RMS displacement = 4.0 × 10^−5^ Bohr.

#### 2.2.2. Excited State Studies of Isolated Dyes

Based on the optimized molecular structures, the optical properties were simulated using time-dependent DFT (TD-DFT) [27] excited-state calculations. Benchmark calculations were carried out on all synthetized dyes with different exchange–correlation XC functionals (XCFs). The theoretical results were compared with experimental data derived from UV-visible absorption spectra. M11 functional demonstrated better performance with the lowest MAE values, therefore, M11/6-311g(d,p)/C-PCM(DMF) level of theory was chosen to simulate the electronic excitations for all the studied compounds. Furthermore, the excited state geometries were optimized and the UV-Vis emission spectra of new dyes were calculated, at the same level of theory, in order to explore the possibility of excitation energy transfer between cosensitizers. All calculations were performed using Gaussian 09 (revision D.01) quantum chemistry software [28]. The electron excitation analysis module, as implemented in Multiwfn 3.8 code [29], was then employed for hole-electron analysis [30] upon photoexcitation besides analyzing quantitatively the intramolecular charge transfer (*CT*) using interfragment charge transfer (IFCT) analysis.

#### 2.2.3. Ground State Studies of Adsorbed Dyes

The efficiency of electron injection, from the new dyes into the *CB* of TiO_2_, was assessed by investigating the interfacial electronic structure of adsorbed isolated dyes on the surface of TiO_2_. Hence, a cluster model of (TiO_2_)_38_ was constructed by appropriately cutting an anatase slab, exposing the majority (101) surface, which was comprised of two layers with 19 Ti atoms on each side. Following the work by Persson et al. [31], appropriate cleaving was achievable by systematic stripping off stoichiometric units (TiO_2_)_n_ from supercell, such that the constructed cluster is stoichiometric, lacking permanent dipole moment, and with high coordination of every constituent atom. The ground state geometries of the bare (TiO_2_)_38_ cluster and of the corresponding dye-adsorbed structures were optimized by the Amsterdam density functional program (ADF) program [32], as integrated into AMS2020.103 software package [33] based on the zero-order regular approximation (ZORA) [34] two-component relativistic Hamiltonian. The ZORA Hamiltonian is the zero-order regular approximation to the Dirac Hamiltonian including a scalar relativistic (SR) and a spin-orbit coupling (SOC) part. The generalized gradient approximation of Perdew-Burke-Ernzerhof (PBE) [35] functional was used in conjunction with Slater-type orbital basis sets [36] TZP/DZP for Ti/H, C, N, O, and S atoms. The considered adsorption mode for carboxyl anchor-based dyes is the bridged homogeneous bidentate mode with both carboxylic oxygens of anchoring group of dye interact with penta-coordinated (5c) Ti sites on two successive rows with proton transferred to nearby surface oxygen (2c) atom. For Pyridyl anchor-based dyes, the monodentate adsorption mode with penta-coordinated (5c) Ti site was adopted.

#### 2.2.4. Electronic Structure of Dimer

For the suggested promising cosensitizer, optimized geometries of monomers were used to construct 50 initial cluster configurations using Genmer tool in Molclus code [37]. Molclus was then employed to invoke MOPAC 2016 software [38] for sequential batch optimization of the 50 configurations using the PM7 [39] (Parametrization method 7) semiempirical quantum mechanics method. Subsequently, Isostat tool in Molclus code was used for sorting the 50 cluster configurations by energy and deleting duplicated structures. Finally, fine optimization of the most suitable lowest energy dimer configuration was performed with M06-2X-D3/6-311G(d,p) level of theory including the D3 dispersion-correction [40] using Gaussian 09 software. The chemical compatibility and stability of the electronic structures of cosensitizers was further investigated by calculating the interaction energies for the heterodimer and performing simple energy decomposition analysis (EDA). The nature of intermolecular interactions between proposed cosensitizers was then studied by none-covalent interaction (NCI) method [41] as supported by Multiwfn 3.8 code along with VMD visualization code [42].

#### 2.2.5. XC-Functionals Assessment

Excitation energies are related to the response of the system to an electromagnetic field; therefore, they depend on how well the virtual orbitals are described. Since complexities of all quantum-mechanical contributions of the many-body problem are cast into the exchange-correlation (XC) functional E_XC_[*ρ*], then predictive power of DFT calculations depends solely on the validity of the chosen approximation to the XCF [43]. Accordingly, UV-Vis absorption spectra for all studied compounds were simulated using 11 different XCFs: the hybrid generalized-gradient approximation (GGA) functional B3LYP [44] with 20% HFX (Hartree–Fock exchange); the range-separated hybrid (RSH) GGA functional CAM-B3LYP [45] (19% HFX at short range and 65% HFX at long range); the RSH meta-GGA functional M11 [46] (42.8% HFX at short range and 100% HFX at long range); the RSH with dispersion effects, ωB97X-D [47] (22% HFX at short range and 100% HFX at long range); the RSH-GGA functional HSE06 [48,49] (25% HFX at short range and 0% HFX at long range); the RSH meta-nonseparable gradient approximation (NGA) functional MN12-SX [50] (25% HFX at short range and 0% HFX at long range); the RSH-NGA functional N12-SX [50] (25% HFX at short range and 0% HFX at long range); the RSH-GGA functional HISS [51,52] (0% HFX at short range, 60% HFX at middle range and 0% HFX at long range); and the meta-GGA hybrid functionals: M06-2X [24] (54% HFX), TPSSh [53] (10% HFX) and τ-HCTH hyb [54] (15% HFX).

**Figure 1:**
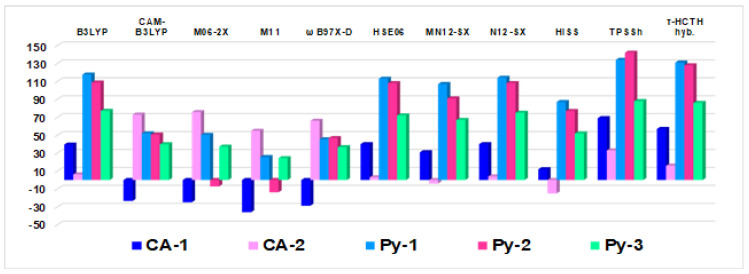
Mean signed deviation (nm) of calculated λ_max_ to experimental λ_max_.

The meta-GGA hybrid functionals were chosen as an increasing order of the percent of Hartree–Fock exchange (HFX%) added to the functional, whereas the RSH functionals were chosen to be belonged to three different categories. Namely the screened-exchange functionals (HSE06, N12-SX and MN12-SX) for which the HFX% decreases monotonically from a finite value (25%) at short interelectronic separations (short range) to zero at long interelectronic separations (long range); the middle-range functional (HISS) for which HFX% increases from zero at short range to a finite value (60%) at medium interelectronic separations and then decreases to zero at long range; the long-range-corrected functionals (M11 and ωB97X-D) for which HFX% increases monotonically from a finite value at short range to 100% at long range; and the CAM-B3LYP functional, for which X increases monotonically with increase in interelectronic separation, but not all the way up to 100% at long range. The mean signed deviation was calculated as: $MSD=\frac{1}{n}\sum_{i=0}^{n}\left(\lambda_{calc}-\lambda_{exp}\right)$, for the absorption maximum wavelength (λ_max_) obtained using the eleven XCFs. While the mean absolute error (*MAE*) was calculated as: $MAE=\frac{1}{n}\sum_{i=1}^{n}\left|\lambda_{calc}-\lambda_{exp}\right|$. As shown in Figure 1, M06-2X as well as the long-range corrected functionals (M11, CAM-B3LYP and ωB97X-D) outperform other functionals in reproducing the excitation energies of pyridine anchor-based set (Py1-Py3) and providing the least *MSD*. Among these functionals, the long-range-corrected RSH meta-GGA functional M11 is the best performing XCF with *MSD* of ca. +26, 14, 24 nm for Py1, Py2 and Py3, respectively. Whereas, for the –COOH anchor-based set (CA1 and CA2), the screened-exchange meta-NGA functional MN12-SX and the middle-range functional (HISS) are the most accurate. This could be attributed to the different types of excitations and different degree of spatial overlap between frontier molecular orbitals. In systems with large rearrangement of charge upon photoexcitation, the asymptotic behavior of M11, M06-2X, CAM-B3LYP and ωB97X-D functionals could lead to a better description of the excitations. In terms of *MAE*, the values were found within a range of 26–78 nm, with following trend: M11 < M06-2X < ωB97X-D < CAM-B3LYP < HISS < MN12-SX < HSE06 < N12-SX < B3LYP < τ-HCTH hyb < TPSSh. For the present two sets of molecules, the most reliable XCF turns out to be M11, with a *MAE* of 26 nm.

#### 2.2.6. Characterization of Electrochemical Properties for Isolated dyes

One of the three factors that determines the overall power conversion efficiency (PCE) of the DSC device is the short-circuit photocurrent density (J_SC_), which can be calculated using the following equation [55]:

(1) $$J_{\mathrm{SC}}=\int^{\;}\mathrm{LHE}\left(\lambda \right)\Phi_{\mathrm{inject}}\eta_{\mathrm{collect}}d\lambda$$

LHE is light-harvesting efficiency at wavelength λ, Φ_inject_ is the quantum yield for the electron injection efficiency, and η_collect_ is the charge collection efficiency which is controllable by the architecture of DSCs only. In contrast, LHE and Φ_inject_ strongly depend on the structural and quantum phenomenon of the dye. The LHE is related to the oscillator strength f of the dye at the maximum absorption wavelength $\left(\lambda_{\max}\right)$, and is expressed as [56]

(2) $$\mathrm{LHE}\left(\lambda \right)=1–10^{-f}$$

The electron injection efficiency Φ_inject_ from the photoinduced excited states of organic dyes into the conduction band (*CB*) of the semiconductor can be related to the free energy change for the electron injection, where $\Phi_{\mathrm{inject}}\propto f\left(-\Delta G_{inj}\right)$. The electron injection driving force (Δ*G_inj_*) can be defined through the following equation [55,57]:

(3) $$\Delta G_{inj}=E_{CB}^{{\mathrm{TiO}}_{2}}-E_{ox}^{*}=E_{CB}^{{\mathrm{TiO}}_{2}}-\left(E_{ox}+E_{0-0}\right)$$

where $E_{ox}^{*}$ and $E_{ox}$ are the oxidational potential of the dye in excited state and ground state, respectively. $E_{CB}^{{\mathrm{TiO}}_{2}}$ is the reduction potential of the *CB* of TiO_2_, in which the experimental value −4.00 eV [58] (vs. vacuum) was used and $E_{0-0}$ is the electronic vertical transition energy at $\lambda_{\max}$. The driving force for efficiency of dye regeneration (Δ*G_reg_*) is calculated via the following equation [59,60]:

(4) $$\Delta G_{reg}=E_{\left(I^{-}/I_{3}^{-}\right)}-E_{ox}$$

In which $E_{\left(I^{-}/I_{3}^{-}\right)}$ is the oxidation potential of the electrolyte (−4.8 eV) [61]. Furthermore, the competing loss pathway (dark current), namely the recombination reaction of the injected electrons in *CB* of TiO_2_ with I_3_^−^ ions in the electrolyte solution can be assessed using the free energies driving force of recombination reaction according to the following equation [59,62]:

(5) $$\Delta G_{rec}=E_{CB}^{{\mathrm{TiO}}_{2}}-E_{ox}\;$$

Accordingly, Equations (2)–(5) are to be employed in this study for estimating the photovoltaic (PV) performance of DSCs employing the new synthesized dyes.

## 3. Results and Discussion

### 3.1. Ground State Geometrical Structures of Isolated Sensitizer

**Figure 2:**
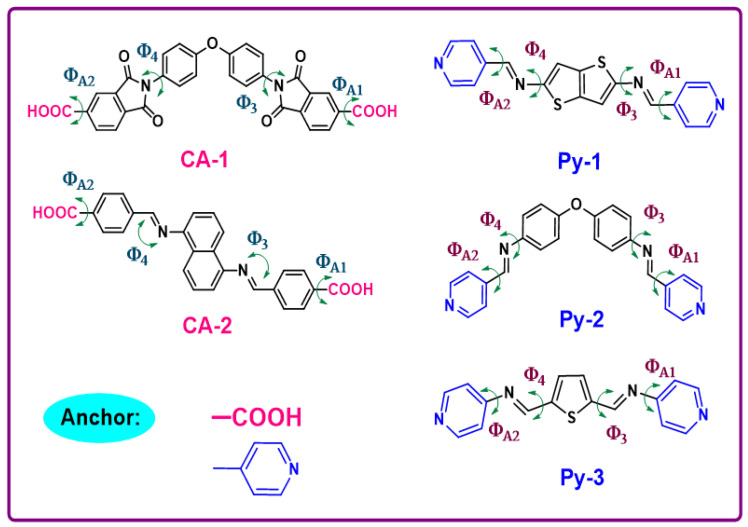
Molecular structures of the new sensitizers with critical dihedral angles positions.

Usually, the conjugation degree of the dye photosensitizer has a substantial impact on the absorption of light and the consequential electronic transitions. Structural attributes, such as the critical twist angles between dye’s fragments, directly disturb the degree of conjugation. The five synthesized dyes are represented in Figure 2 with displaying the positions of critical dihedra angles, where their calculated values are listed in Table 1. The dyes are grouped into two sets of molecules according to the type of anchoring group; namely CA set with the –COOH anchor and Py set with pyridine ring as an anchor. The optimized geometries of the free dyes are displayed in Figure S1 (see the Supplementary Materials).

Comparing the dyes with –COOH anchor, all geometries deviate from the planarity by ca. 29–50° due to the steric repulsion between rings, where the diimide CA-1 dye obtained a bent shaped geometry as the rings in diphenyl ether tend to be on planes perpendicular to each other. Considering the dyes with pyridine anchor, Py-1 dye has significantly enhanced the planarity and can be considered the most planar structure among the studied dyes. Similar to CA-1 dye, Py-2 dye showed a bent shaped geometry with the two rings of diphenyl ether are in planes at right angles to each other.

Comparing the significant increment of Φ_A1_ and Φ_A2_ twist angles of Py-3 (ca. 45°) than Py-1 (ca. 2°), where they have same pyridine anchor but with different attachment points to imine (–CH=N–) linkage, namely imine carbon and imine nitrogen of Py-1 and Py-3, respectively. It is obvious that improved conjugation of the π-cloud of dithiophene moiety with the lone pair of imine N in Py-1 dye enforces the planarity of its conjugated system. This is reflected in the bond lengths of thiophene-C–N(imine) and thiophene-C–C(imine) of Py-1 and Py-3 dyes, which were found to be 1.37 Å and 1.45 Å, respectively.

According to the basic principle of DSCs, after excitation of the dye by incident light, an oxidized state of the dye is formed by electron injection into the conduction band (*CB*) of the anode (here TiO_2_). Since maintaining the planarity between the anchoring group and the molecular plane is critically required for the ease of charge flow and electron injection into the *CB* of TiO_2_, then we could argue that Py-1 and Py-2 dyes will show enhanced electron injection efficiency, according to their lowest values of Φ_A1_ and Φ_A2_ dihedral angles.

### 3.2. Frontier Molecular Orbitals Analysis

**Figure 3:**
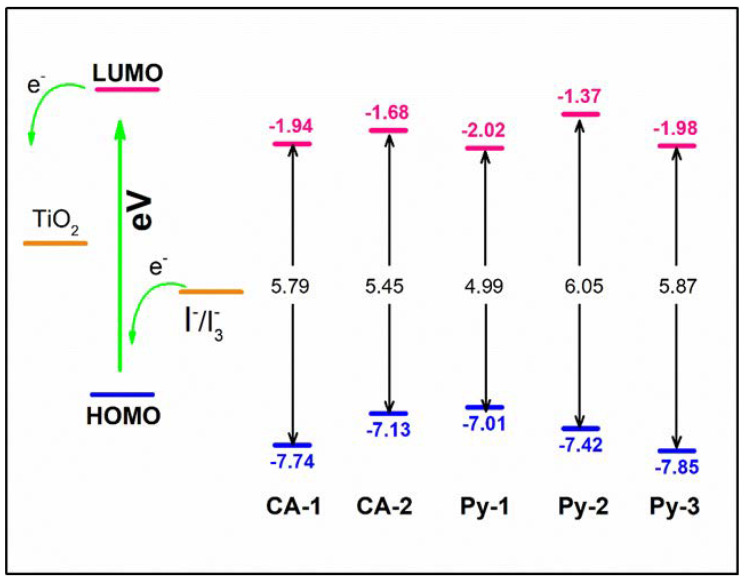
Energy level diagram of the HOMO and LUMO for all dyes, TiO_2_, and the electrolyte.

For efficient operation of DSC, the lowest unoccupied molecular orbital (LUMO) of the dye should be higher than the *CB* (−4.00 eV) of TiO_2_, with at least 0.2 eV [63], to guarantee efficient electron injection. Similarly, the highest occupied molecular orbital (HOMO) of the dye should lie below the energy level (−4.8 eV) of the iodine/triiodide ($I^{-}/I_{3}^{-}$) redox electrolyte for effective regeneration of the oxidized dyes. The energy levels involving E_HOMO_ and E_LUMO_ and their energy gaps of all new dye molecules are shown in Figure 3, together with the energies of $I^{-}/I_{3}^{-}$ redox potential and *CB* of TiO_2_ for a direct comparison. It can be seen that the calculated LUMO energy levels of all dyes range from −2.02 to −1.37 eV, which are about 1.98 to 2.63 eV higher than the *CB* of TiO_2_. Accordingly, thermodynamically favored electron injection reactions can be predicted for both CA and Py dyes. Additionally, the HOMO energy levels ranging from −7.85 to −7.01 eV are significantly lower than the electrode potential of the redox couple (−4.8 eV), implying that thermodynamically favored regeneration reactions are expected for all dyes. Evidently, E_HOMO_ and E_LUMO_ of all studied dyes meet the basic energetic requirements for operating DSCs; however, the HOMO level of Py-3 shifts toward more negative value (−7.85 eV) suggesting easier regeneration of the oxidized dye with greater driving force, when compared to other dyes.

Furthermore, Figure 3 vividly shows that Py-1 dye possesses the smallest HOMO–LUMO energy gap (Eg) of 4.99 eV. This can be attributed to the inclusion of fused thienothiophene heterocycle, which increases the conjugation length thus, causes lower energy gaps and eases the photoexcitation of dye molecules. This result is consistent with the previously detected enhanced planarity of Py-1 dye. Since strong acceptor decreases the E_LUMO_, then one can assume that pyridine rings of Py-1 and Py-3 would have greater accepting power according to their lowest (−2.02 and −1.98 eV) values of E_LUMO_, which is beneficial for operating DSC.

**Figure 4:**
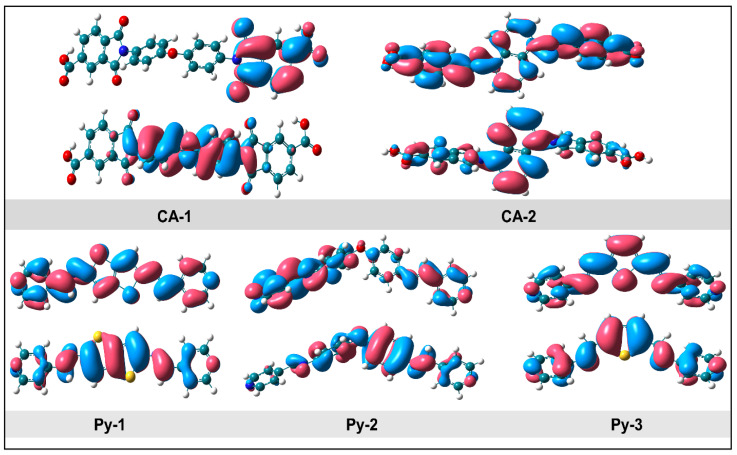
Contour plots of the HOMO and LUMO of isolated dyes. In all the cases the isovalue is set to 0.02. HOMO level is depicted below and LUMO level is depicted above for each dye design.

The contour plots of the FMOs for the six dyes are depicted in Figure 4. Considering CA-1 and Py-2 dyes, the HOMO electron density is mostly located on the diphenyl ether unit, whereas the LUMO is predominantly spread to the acceptor units, which satisfies the requirements of the molecular orbital for charge separation. Distinct hole-electron separation could not be estimated for CA-2 and Py-3 dyes as the electron densities of their FMOs are nearly distributed throughout the entire molecular backbones. Concerning Py-1 dye, its structural attributes and pattern of FMOs distributions can suggest promising injection of excited charge into *CB* of TiO_2_, particularly for increased localization of LUMO on the pyridine anchor as conjugated with the imine group. The same conclusion was previously drawn for some squarine dyes with planar structure and great π–electron density conjugation along molecular backbone [64].

### 3.3. Photoabsorption Spectra of Free Dyes

**Figure 5:**
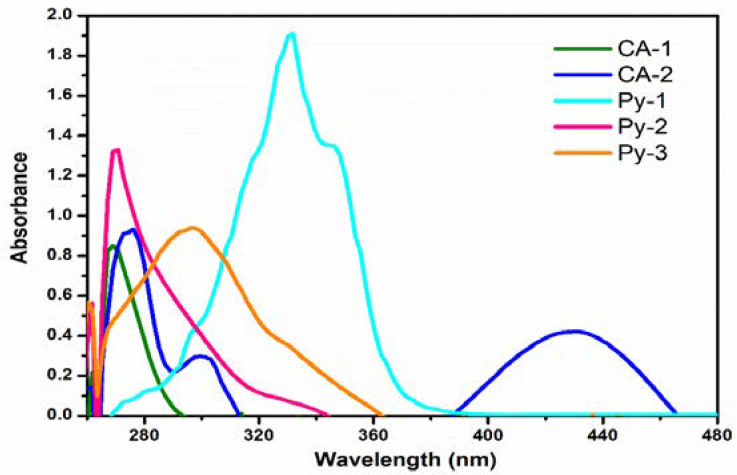
Experimental UV-Vis spectra of the synthesized dyes measured in DMF solvent.

The light absorption behavior of the cosensitizer is a crucial factor for evaluating its photophysical properties and determining its ability to achieve complementarity of absorption spectrum. Figure 5 depicts the experimental UV-Vis spectra of the synthesized UV-dyes, measured in DMF solvent. Table 1 lists the optical data obtained from TD-DFT calculations along with the experimental measurements of λ_max_. The dominant character of the transitions was assigned according to the relocations of the FMOs (Figure 4 and Figure S2), in which each excited state is characterized by either intramolecular charge transfer (ICT), π–π* local excitation (LE) or hybridized local excitation–*CT* (HLCT) state. HLCT state occurs when a localized excitation is present close to the *CT* excitation [65].

The experimental UV-Vis spectra (Figure 5) show that all dyes exhibit an absorption band covering the mid-UV/near-UV region (269–330 nm) of solar spectrum. The relative bathochromic shift of Py-1 dye is signifying the previously calculated lower energy gab between the frontier molecular orbitals due to boosted planarity and conjugation length. Additionally, a hyperchromic shift is observed for Py-1 dye which is attributable to the incorporation of fused thienothiophene heterocycle. Judicious choice of fused conjugation unit of Py-1 dye is expected to enhance the light-harvesting capability and ultimately the higher PCE. The calculated electronic absorptions (Table 2) agree with the experimental observations in reproducing the relative red-shifted λ_max_ of Py-1 dye.

For CA-2, Py-1 and Py-3 dyes, the maximum light absorption at λ_max_ corresponds to first electronic transition S0→S1, with HOMO→LUMO being the major (64–87%) contributing molecular orbitals. Since the spatial distributions of HOMO and LUMO for these dyes reflects electron delocalization over the entire molecule, with greater localization of LUMO on the anchoring groups of Py-1 and CA-2. Thus, transitions of Py-1 and CA-2 are characterized by hybridized ICT-local π–π* excitation (HLCT), whereas for Py-3, the local π→π* excitation is the primary transmission mode.

The main absorption peak of CA-1 dye with λ_max_ at 270 nm is mainly made up of S12 state. This predominantly corresponds to HOMO→LUMO + 5 (45%) and HOMO→LUMO + 2 (21%), where HOMO and LUMO + 5 orbitals are distributed over the same diphenyl ether unit while LUMO + 2 is localized on isoindoline-1,3-dione. Consequently, S0→S12 electronic transition can be assigned as HLCT. Similarly, S5 excited state of Py-2, involves local electron excitation in the imine and pyridine units (HOMO-8→LUMO + 1) addition to intramolecular *CT* from diphenyl ether to imine and pyridine (HOMO→LUMO) i.e., HLCT transition. The larger values of oscillator strength (f) is regarded to larger values of transition electric-dipole moment, which means that S0→S1 transition of Py-1 is a strongly electric-dipole allowed transition. Overall, optical properties of all new UV-absorbing dyes suggest the obtainability of complementarity of absorption spectrum when combined with the NIR-absorbing VG20 reference dye, particularly, Py-1 dye with boosted light-harvesting capability.

### 3.4. Emission Characteristics

Assessing the nature of the fluorescent state of photosensitizing dyes is one of the foremost tasks, since long lifetime of stable fluorescent state endows a substantial role in the electron mobilization process in DSCs [66]. Therefore, the linear response (LR) TD-M11/CPCM calculations have been carried out to reveal the kinetics of decays of the excited states. The radiative lifetime was then calculated through the Einstein transition model [67] $\tau_{flu}=c^{3}/\left(2\times {\left(E_{flu}\right)}^{2}\times f\right)$, in which *c* stands for speed of light in au, *E_flu_* is the fluorescence transition energy in cm^−1^ and f is the oscillator strength for the S0←S1 transition. Table 3 lists the fluorescence energies *E_flu_* (eV), fluorescence wavelengths λ_em_ (nm), oscillator strengths (f) and radiative lifetimes of the first excited states (S1).

**Figure 6:**
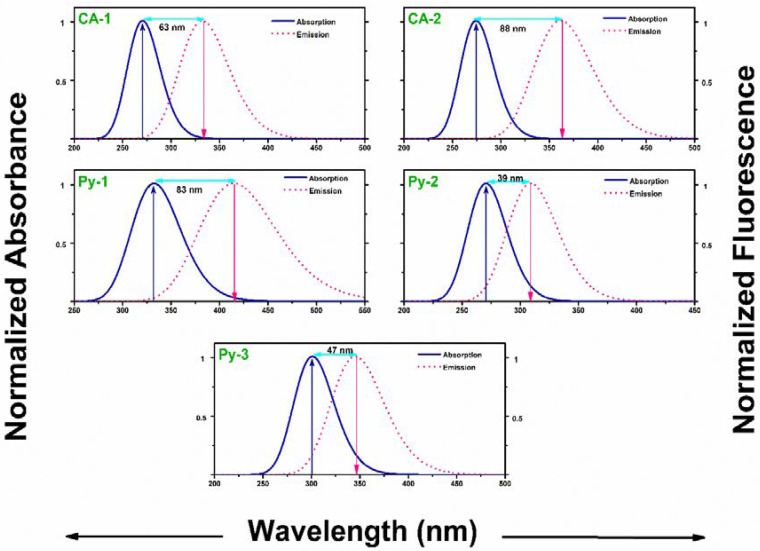
Absorption and emission spectra of the UV-dyes along with the calculated Stokes shift.

Figure 6 displays the computed emission spectra of the synthesized dyes along with a comparison with the corresponding experimental absorption maxima and calculated Stokes shift (red shift) of the fluorescent state. Notably, emission spectra of the studied dyes showed emission peaks in the range of 309–415 nm. The optimized geometries of emission states are shown in Figure S3 (see the Supporting Information). The optimized structures show that Φ_3_ and dihedral angle of CA-1 has decreased by ca. 25° in comparison to the ground-state. Similarly, an enhanced planarity has been obtained for Py-3 dye with ca. 18° lower Φ_A1_ and Φ_A2_ relative to the ground-state geometry.

A perpendicularly twisted terminal benzylidene carboxylic acid group and pyridine group to the molecular plane, has been found in CA-2 and Py-1, respectively. Accordingly, emission peak of their fluorescence spectrum could be attributed to a twisted, relaxed excited state. The calculated larger values of the Stokes shift of CA-1, CA-2 and Py-1 dyes lying in the range of 63−88 nm are indicative of their structural flexibility upon photoexcitation. For CA-1 dye, despite showing the longest lifetime (3.5 ns) for fluorescence process, its negligible value of oscillator strength (ca. 0.0004) indicates that no emission exists. On the other hand, the results revealed that Py-1 and CA-2 dyes showed the largest emission peaks and Stokes shifts with the lowest radiative lifetime.

Since the emitted photons can be trivially reabsorbed if the absorption and fluorescence spectra of the cosensitizers overlap [68], the possibility of excitation energy transfer from any of the new synthesized UV-dyes to the proposed VG20 cosensitizer is not expected for the reason that there is no overlapping between absorption spectrum of VG20 (826 nm) and fluorescence spectra of the UV dyes (309–415 nm).

### 3.5. Intramolecular Charge Transfer Characteristics

**Figure 7:**
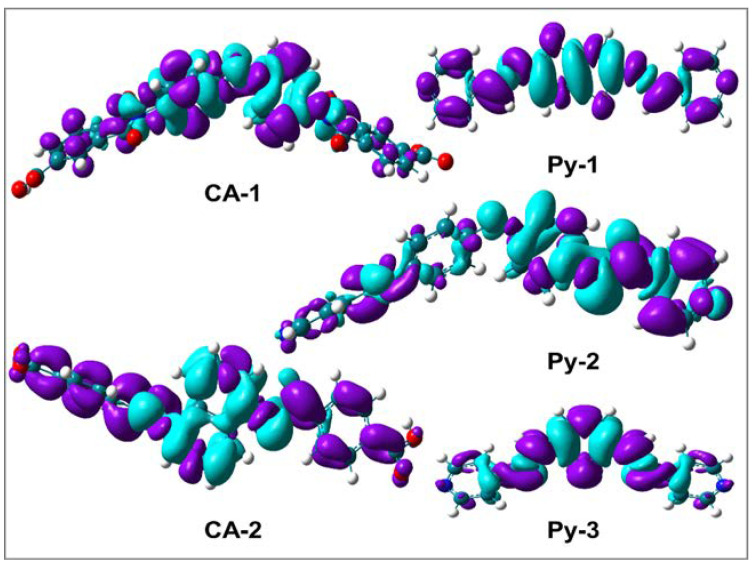
Isosurface of charge density difference (CDD) map. Purple and cyan correspond to electron and hole.

Here, direction of intramolecular charge transfer (*CT*) that is advantageous to the interfacial electron injection, is examined using Hole-electron analysis module [30] as implemented in Multiwfn 3.8 code. Therein, electron excitations are designated as a process from hole to electron; therefore, graphical analysis and quantitative descriptors are obtainable by this approach. Moreover, the number of excited electron, Δq (|*e*^−^|), received by the anchoring groups was calculated using interfragment charge transfer (IFCT) method. The results are tabulated in Table 4. The charge density difference (CDD) between excited state and ground state is estimated as: $\Delta \rho \left(r\right)=\rho^{ele}\left(r\right)-\rho^{hole}\left(r\right)$. Figure 7 depicts the visualized isosurface of CDD maps for the excited states studied in Table 2. In which purple and cyan colors are representing the electron and hole distributions, respectively.

Quantitative descriptors of *CT* involve *D_CT_* (A°), the *CT* distance; t-index (A°), the charge separation degree; *E_C_* (eV), the Coulomb attraction energy; and Sr (au), the hole-electron overlap index.

The charge transfer distance, *D_CT_*, is calculated as: $D_{CT}=\sqrt{{\left|X_{e}-X_{h}\right|}^{2}+{\left|Y_{e}-Y_{h}\right|}^{2}+{\left|Z_{e}-Z_{h}\right|}^{2}}$ to represent the spatial distance between C_hole_ and C_ele_ centroids in *X*, *Y* and *Z* direction. Where *X*, *Y* and *Z* stand for the three-dimensional coordinates of centroids. For example, *X_e_* represents the electron density at *X* orientation with the detailed expression: $X_{e}=\int^{\;}x\rho^{ele}\left(r\right)dr$ with *ρ^ele^*(*r*) representing the electron density distribution and *x* being the X component of position vector *r*. The charge separation degree in the direction of *CT*, t-index, is calculated by: $t\;\mathrm{index}=D_{CT}-H_{CT}$, in which *H_CT_* is the average degree of spatial extension of hole and electron distribution in *CT* direction. This means that clear separation of hole and electron distributions is assigned by positive value of t-index. The Coulomb attraction energy, *E_C_* (eV), is calculated as follows:

$E_{C}=\iint^{\;}\frac{\rho^{hole}\left(r_{1}\right)-\rho^{ele}\left(r_{2}\right)}{\left|r_{1}-r_{2}\right|}\;dr_{1}\;dr_{2}$, which estimates the required energy for dissociating the photoinduced exciton. The hole-electron overlap index (*Sr*) is calculated as: $S_{r}=\int^{\;}\sqrt{\rho^{hole}\left(r\right)\;\rho^{ele}\left(r\right)\;}dr$, for comparing the overlapping extent of hole and electron. Hence, the theoretical upper limit of *S_r_* index is 1 au implying that almost all part of hole and electron distributions are perfectly matched. As presented in Figure 7, photoexcited electron accumulation is clearly observed in anchoring groups of all dyes, except for CA-1 and Py-3. For CA-1, contribution of photoexcited electron to –COOH anchoring group is negligible, which can be confirmed by the Δq value of 0.001 |*e*^−^|. Similarly, the isosurface of CDD between S0 and S1 of the Py-3 dye, spread over the molecule with alternative presentation of the positive part and negative part presented alternatively, assuring the previously predicted local π–π* excitation of S0→S1 transition.

Generally, it can be seen that hole–electron distribution displayed in the CDD maps (Figure 7) is supported by the numerical calculated data tabulated in Table 4. After photoexcitation, the propitious separation of molecular exciton requires smaller *E_C_* to overcome. Consequently, the intramolecular electron−hole (exciton) pair is easier to separate in Py-2 (2.607 eV), whereas the predicted barrier reaches the maximum (4.430 eV) for Py-3, as presented in Table 4. By examining the obtained large values of *S_r_* (>0.50 A°), the negative values of t-index and small values of *D_CT_*, one can conclude that the charge separations are not perfect for all the studied dyes. Nevertheless, according to the IFCT analysis for assessing the amount of charge received by the anchoring group, Py-1 dye with Δq of ca. 0.2 |*e*^−^| is expected to have the largest amount of injected electron into the *CB* of TiO_2_, consistently with the order of red-shifted maximum absorption wavelength and improved ability of incident light harvesting. Additionally, the unidirectional intramolecular charge transfer (*CT*) of the S0→S1 electronic transition that is advantageous to the interfacial electron injection, can be observed in CA-2 and Py-1 dyes.

### 3.6. Electrochemical Performance of the Dyes

The ability of a dye to capture incident light (photon) upon photoexcitation, is one of the most crucial factors affecting the overall power conversion efficiency (PCE) of DSC. It can be evaluated by calculating light harvesting efficiency (LHE) according to Equation (1). The estimated values of LHE along with the computed critical energy parameters of the present study are tabulated in Table 5. Since values of oscillator strength (f) obtained from TD-DFT calculations could not reproduce the same trend of experimentally measured absorbance intensities.

Explicitly, the experimental measurements of absorbance showed the following trend: Py-1 (1.910 au) > Py-2 (1.32 au) > Py-3 (0.94 au) ≅ CA-2 (0.93 au) > CA-1 (0.85 au) while theoretically, the intensity of peaks shows the trend CA-1 > Py-1 > Py-2 > Py-3 > CA-2, as presented in Table 2. Therefore, a correct estimation of absorbance intensities obtained from experimental measurements will be used to accurately assess light harvesting efficiency of studied dyes, rather than theoretical attained trend. Accordingly, Py-1 dye recorded the highest LHE value of 0.971 which is consistent with the high perturbation of the charge distribution of the extended conjugation length along molecular backbone. This is reflected in the enlargement of the transition dipole moment of the excited state because the large hole−electron overlap is the precondition of the large transition dipole moment [69]. Conversely, the weakest light harvesting ability is predicted for CA-1 dye.

The thermodynamical feasibility of electron injection and regeneration processes are evaluated by calculating the driving forces, Δ_Ginj_ and Δ*G_reg_*, moreover evaluation of Δ*G_rec_* according to Equations (3)–(5). It is obvious that the negative values of Δ*G_inj_* for all dyes imply the spontaneity of the electron flow from LUMO of the dye into the *CB* of TiO_2_ semiconductor. Similarly, negative values of Δ*G_reg_* signify the spontaneity of the electron transfer from the reduce redox species in the electrolyte to the oxidized state of dye, i.e., thermodynamically favored regeneration processes. CA-1 dye has the highest negative value (−1.56 eV) of Δ*G_inj_*, implying that it has the greatest energetic driving force of the electron injection. Py-3 dye recorded the highest negative value (−3.05 eV) of Δ*G_reg_* displaying a very promising driving force for regeneration process. Generally, more negative values of Δ*G_reg_* than Δ*G_inj_* values indicates that dye regeneration dynamics is more stable than electron injection [55]. The positive values of Δ*G_rec_* for all dyes implies that the competing loss electron recombination reaction is nonspontaneous processes in all dyes, which is knowingly crucial for operating DSC.

### 3.7. UV-Dyes Adsorbed on (TiO_2_)_38_ Cluster

**Figure 8:**
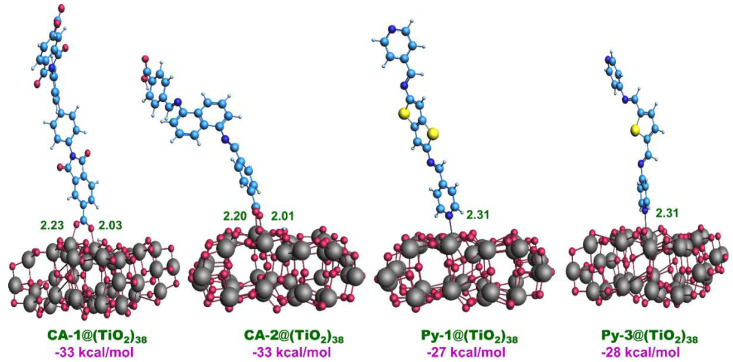
Optimized geometrical configurations of dyes adsorbed onto the surface of (101) anatase (TiO_2_)_38_ cluster along with calculated adsorption energies (kcal/mol) and the distance from the receptors to the surface of TiO_2_ (Ti–O and Ti–N, Å).

To gain deep insight into the interfacial electronic injection process, electronic coupling of the synthesized dyes as adsorbed onto the surface of TiO_2_ semiconductor is to be simulated. The equilibrium interface geometries are presented in Figure 8. Except for Py-2 dye, its optimized geometry did not converge using the same convergence criteria applied for other dyes so it will be excluded from this analysis. So as to assess the robust interaction and electronic coupling between TiO_2_ surface and the dyes, the adsorption energy (*E_ads_*) of the studied systems was calculated according to the equation: $E_{ads}=E_{\left(\mathrm{dye}@{\mathrm{TiO}}_{2}\right)}-\left(E_{\mathrm{dye}}+E_{{\mathrm{TiO}}_{2}}\right)$ where $E_{\mathrm{dye}}$, $E_{{\mathrm{TiO}}_{2}}$ and $E_{\left(\mathrm{dye}@{\mathrm{TiO}}_{2}\right)}$ are energy of isolated dye, bare (TiO_2_)_38_ cluster and dye@(TiO_2_)_38_ complex, respectively. Critical geometrical parameters of the optimized configurations and the calculated adsorption energies for dye@(TiO_2_)_38_ are also reported in Figure 8.

The calculated negative adsorption energies *E_ads_*, lying in the range of −27 to −33 kcal/mol, suggest that adsorption of all dyes is stable and spontaneous. The geometric features of the optimized structures reveal that the dyes stay vertically above the TiO_2_ cluster surface, with the maximum inclination observed in CA-2 dye. This may allow for relatively faster charge recombination and lower the device efficiency.

The interacting distance between the carbonyl oxygen and the Ti-atom of CA-1 and CA-2 dyes are 2.23 and 2.20 Å, whereas for Py-1 and Py-3 dyes, distance between pyridyl nitrogen and Ti is ca. 2.31 Å. These critical geometrical parameters support the formation of stable complexes of tightly adsorbed dyes onto the surface of TiO_2_. Comparing the geometrical parameters of isolated dyes (Table 1) and dyes adsorbed onto TiO_2_ surface, the twist angle (Φ_A1_) of anchoring groups increased to 28°, 25° and 0.2° for CA-1, CA-2 and Py-1 dyes signifying the steric hindrance variations induced by the adsorption. While Φ_A1_ of adsorbed Py-3 remains as the same (46°) as that of isolated dye. Again, the smallest value (0.2°) of Φ_A1_ of Py-1, predicts that the photoexcited electrons can be efficiently injected into the TiO_2_ film.

**Figure 9:**
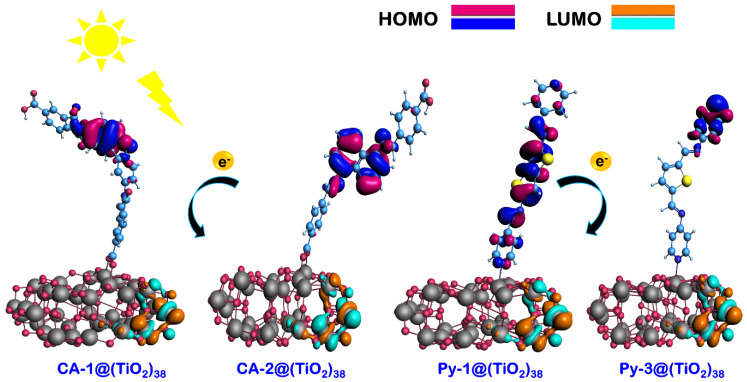
Contour plots of the HOMO and LUMO of the dye adsorbed onto the surface of (101) anatase (TiO_2_)_38_ cluster.

The spatial distribution of FMOs of the dyes @(TiO_2_)_38_ cluster model with 101 surface was also calculated and displayed in Figure 9. Compared with those (Figure 4) of CA-1, CA-2 and Py-3 dyes, the significant difference in HOMO localized distributions for the dye@TiO_2_ systems support the electronic structure variations resulting from electronic coupling between the dyes and TiO_2_ cluster. Alternatively, electron distribution of LUMO resides in the TiO_2_ cluster in all the studied systems. This relocation of electron density distribution suggests the charge separation at the dye/TiO_2_ interface that is favorable for increasing the electron-transfer rate and enhancing the photoelectric properties.

All told, the UV-selective organic Py-1 dye (332 nm) can be proposed for potential cosensitization for the NIR-selective VG20 (825 nm) dye-sensitized solar cell. Among the five synthesized UV-selective organic dyes, Py-1 dye exhibits the most advantageous structural attributes for sensitization, has the longest conjugation length; attains the basic required energetics of operating DSCs, possesses the smallest HOMO–LUMO energy gap and shows the greatest accepting power of pyridine anchoring group. Additionally, it has the most intense emission band with large Stokes shift, demonstrates stable and spontaneous adsorption onto surface of TiO_2_, has an accepted distribution of FMOs electron densities either as free dye or adsorbate, and displays thermodynamical spontaneity of electron injection and dye regeneration processes. Most importantly, Py-1 dye achieves the complementarity of absorption spectrum with the largest hyperchromic shift and a broad absorption band in the near-UV subregion corresponding to S0→S1 electronic transition and HOMO→LUMO as dominant configuration. In addition to the predicted largest amount of charge received by the anchoring group, the Py-1 dye absorption band was assigned as hybridized *CT* and local π–π* excitation (HLCT), which could generate synergistic enhancement effects for the *CT* process [70,71].

Furthermore, we chose the Py-1 dye because it features the highest value of measured absorptivity and computed light harvesting efficiency, therefore, the performance of the VG20-based transparent cell can be improved, as thinner film is often employed for high molar absorptivity dyes. The small molecular size of Py-1, relative to VG20 dye, can also minimize the dyes competitive adsorption issues. Above all, the UV-absorber Py-1 and the NIR-absorber VG20 have pyridine and –COOH as anchoring groups, respectively. This is a known strategy to overcome the competitive adsorption due to cosensitization, namely by combining dyes with pyridyl and carboxyl anchoring groups that respectively prefer Lewis- and Bronsted-acidic anchoring sites on TiO_2_. The advantage of the optical complementarity and the different TiO_2_-adsorption-site selectivity of Py-1 and VG20, can be accomplished accordingly.

### 3.8. Electronic Structure of Py-1/VG20 Cosensitizer

**Figure 10:**
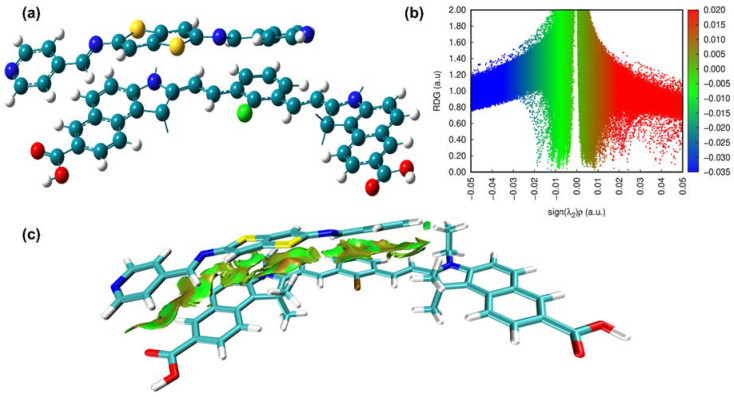
(**a**) Optimized geometry. (**b**) Scatter map of RDG versus electron density *ρ*(*r*) multiplied by the sign of second Hessian eigenvalue λ_2_, (**c**) RDG isosurface map for the Py-1/VG20 dimer.

Electronic incompatibility of cosensitizers is one of the foremost reasons of depleting the photovoltaic (PV) performance of DSCs upon cosensitization. Consequently, the electronic structure of the proposed cosensitizer Py-1/VG20 was calculated, in order to inspect if they are chemically compatible with each other. The obtained equilibrium geometry of Py-1/VG20 dimer is displayed in Figure 10a. It was found that the interlayer distance is ca. 3.9 Å of superimposed Py-1/VG20 structure.

Quantitative assessment of the stability of dimer involves calculation of total interaction energy Δ*E*_tot_ followed by simple energy decomposition analysis (EDA). Calculated value of Δ*E*_tot_ is −38.07 kcal/mol, was obtained via the expression ${\Delta E}_{\mathrm{tot}}=E_{dimer}-\sum_{i}E_{i}^{frag}$. In simple EDA, the total energy variation of forming a dimer is decomposed as: ${\Delta E}_{\mathrm{tot}}={\Delta E}_{\mathrm{orb}}+{\Delta E}_{\mathrm{steric}}+{\Delta E}_{\mathrm{disp}}$. Here, Δ*E*_disp_ is the dispersion interaction (vdW) energy evaluated as ${\Delta E}_{\mathrm{disp}}=E_{\mathrm{tot}}^{\mathrm{DFT}-D3}-E_{\mathrm{tot}}^{\mathrm{DFT}}$, in which the D3 Grimme’s correction is to account for the dispersion energy that is missing in DFT energy. It was found that Δ*E*_disp_ = −5.012 kcal/mol, which means the dispersion weak intermolecular attractive interaction stabilizes the formation of Py-1/VG20 dimer. The steric energy (${\Delta E}_{\mathrm{steric}}$) is the sum of the electrostatic energy, the exchange-correlation energy and the exchange-repulsion (Pauli) term. For the studied dimer, ${\Delta E}_{\mathrm{steric}}$ was found to be −17.68 kcal/mol, implying that electrostatic energy possesses high attractive contributions, since the Pauli term is invariably positive corresponding to the repulsive interaction between the electrons in occupied orbitals of monomers. Calculated ${\Delta E}_{\mathrm{orb}}$ of −15.38 kcal/mol is the orbital interaction energy between the monomers implying a strong attraction originated from the overlap of orbitals between Py-1 and VG20 monomers and stabilizes the formation of the dimer.

Overall, the total interaction energy, Δ*E*_tot_ of −38.07 kcal/mol, indicates that the formation of Py-1/VG20 dimer is favored by the steric, electrostatic, as well as orbital interactions. Figure 10b depicts the plot of reduced density gradient (RDG) versus sign(λ_2_)*ρ*, the sign of second Hessian eigenvalue (λ_2_) multiplied by electron density *ρ*(*r*). Therein, electron density *ρ*(*r*) defines the strength of interactions; namely low *ρ*(*r*) regions are related to the dispersion or vdW weak interactions (green dots) and high *ρ*(*r*) regions related to stronger interactions, either stabilizing attractive (λ_2_ < 0) such as H-bonds (blue dots) or destabilizing repulsive (λ_2_ > 0) such as steric (red dots) interactions. As shown in Figure 10b, the spike at the negative part with low-density (*ρ*(*r*) ≈ 0), low-gradient (RDG < 0.5 au) approves the calculated stabilizing vdW attraction energy Δ*E*_disp_ of −5.012 kcal/mol. Additionally, low-gradient (RDG < 0.5 au) blue dots at sign(λ_2_)*ρ*(*r*) of ca. −0.025 au indicates the presence of the stabilizing H-bonding interaction in the studied dimer.

Visualization of the physical origin of the calculated noncovalent interactions is attainable by observing RDG isosurface map, Figure 10c. Therein, the isosurface is colored on a blue-green-red scale according to values of sign (λ_2_)*ρ* function. The intramolecular interactions were screened out for clear displaying of intermolecular interactions only. Despite the existence of low-gradient spikes (red dots) in the positive region of Figure 10b, demonstrating the occurrence of destabilizing steric repulsion, the lack of red isosurface extending between two monomers in Figure 10c implies that these steric effects are exhibited within the rings. The green isosurface in Figure 10c shows clearly the extending weak vdW attractive interaction regions between Py-1 and VG20 monomers. Considering the calculated low destabilizing energy and the high stabilizing orbital, vdW and electrostatic attractive interactions of the studied dimer, we could argue that Py-1 and VG20 dyes are chemically compatible and this dimer is applicable for cosensitization in DSCs.

## 4. Conclusions

In this work, novel diimide and Schiff bases UV-absorbers have been synthesized by introducing carboxyl and pyridyl anchoring groups to be applied in transparent dye-sensitized solar cells. UV/Vis absorption spectra of solution-based free dyes were measured experimentally. A systematic ground state and excited state investigation was subsequently carried out via DFT and TD-DFT approaches. The dye@TiO_2_ interface was examined via further in silico calculations. Energetics of the studied dyes, either isolated or adsorbed onto TiO_2_ surface, addition to the optical properties and estimated photovoltaic performance, showed that they function well in in DSCs. The pyridyl-containing Py-1 dye, with thienothiophene core, unquestionably outperforms the other studied dyes. In particular, exhibiting the most advantageous structural attributes for sensitization with the most conjugated fragments. Its pyridine anchor exerted the greatest accepting power thus, received the largest amount of calculated transferred charge. Moreover, Py-1 showed an increase of measured absorption and calculated emission intensities with large Stokes shift. This high absorptivity boosts the light harvesting efficiency, which is crucially required in transparent DSCs incorporating sufficiently thin layer of TiO_2_ in order to not be seen. Accordingly, the UV-absorber Py-1 was tested for cosensitization of the NIR-absorber VG20-C_2_. The electronic structure of the Py-1/VG20 dimer was examined via calculated interaction energy, energy decomposition analysis, and reduced density gradient maps. Results showed the chemical compatibility of the proposed cosensitizers with low destabilizing repulsive interactions moreover high stabilizing orbital, vdW and electrostatic attractive interactions. This is in addition to the small molecular size of Py-1, relative to VG20 dye, which is critically needed to minimize the problematic dyes competitive adsorption. Above all, the judicious choice of Py-1 as a cosensitizer for VG20 follows the known strategy of combining cosensitizers with pyridyl and carboxyl anchoring groups for respectively selecting Lewis- and Brønsted-acidic different anchoring sites on TiO_2_. As a consequence, this study suggests the promising application of UV-absorber Py-1 along with the NIR-absorber VG20 in transparent dye-sensitized solar cells of higher power conversion efficiency.

## Figures and Tables

**Table 1:** Calculated Critical Dihedral Angles of New Sensitizers at Ground State (S0) Geometry.

	CA-1	CA-2	Py-1	Py-2	Py-3
Φ_A1_	24	23	0.09	2	45
Φ_A2_	24	23	0.02	2	45
Φ_3_	50	46	0.2	37	1
Φ_4_	49	46	29	40	1

**Table 2:** Experimental and calculated TD-DFT Excitation Energies (eV, nm), Oscillator Strengths (f), Compositions in Terms of Molecular Orbital Contributions, and Dominant Character of the Transitions.

Dye	State	Exp λ_max_	λ_max_	E (eV)	f	Major Contribution	Dominant Transition
CA-1	S0→S12	270	234	5.30	1.74	H→L + 5 (45%); H→L + 2 (21%)	π–π* (LE)
CA-2	S0→S1	276	332	3.72	0.85	H→L (64%); H-1→L + 1 (11%)	π–π* (LE)+ ICT (HLCT)
Py-1	S0→S1	332	370	3.35	1.53	H→L (86%); H-2→L + 1(5%)	π–π* (LE)+ ICT (HLCT)
Py-2	S0→S5	269	255	4.86	1.17	H-8→L + 1 (23%); H→L (12%)	π–π* (LE)+ ICT (HLCT)
Py-3	S0→S1	298	320	3.87	1.08	H→L (87%); H-1→L + 1 (6%)	π–π* (LE)

**Table 3:** Calculated TD-M11 fluorescence energies *E_flu_*, fluorescence wavelengths λ_emi_, oscillator strengths (f) and radiative lifetimes.

Dye	*E_flu_* (eV)	λ_max_ (nm)	f	$\tau_{flu}\;(\mathrm{ns})$
CA-1	3.732	332	0.0004	3.548
CA-2	3.428	362	0.9238	0.002
Py-1	2.988	415	1.4261	0.002
Py-2	4.013	309	0.3477	0.004
Py-3	3.590	345	0.0416	0.037

**Table 4:** Charge transfer descriptors and Δq, the net number of charge transferred to the anchor.

Dye	*D_CT_*	t-Index	*E_C_*	*S_r_*	$\Delta q\;(\left|e^{-}\right|)$
CA-1	0.418	−1.022	2.998	0.649	0.001
CA-2	0.678	−0.575	3.211	0.572	0.050
Py-1	0.648	−0.922	3.702	0.598	0.199
Py-2	1.064	−3.440	2.607	0.596	0.178
Py-3	0.172	−1.102	4.430	0.746	0.013

**Table 5:** Estimated light harvesting efficiency (LHF) and critical energy parameters (in eV).

Dye	LHE *^a^*	$E_{ox}^{*}$	Δ*G_inj_*	Δ*G_reg_*	Δ*G_rec_*
CA-1	0.792	−2.44	−1.56	−2.94	3.74
CA-2	0.820	−3.41	−0.59	−2.33	3.13
Py-1	0.971	−3.66	−0.34	−2.21	3.01
Py-2	0.911	−2.56	−1.44	−2.62	3.42
Py-3	0.823	−3.98	−0.02	−3.05	3.85

*^a^* LHE calculated with corrected values of oscillator strength according to relative values of the measured absorbance.

## Data Availability

Raw data are available upon request.

## Supplementary Materials

The following are available online. Figure S1: optimized geometries of the ground state of the studied dyes, Figure S2: major contributing molecular orbitals in the studied electronic transitions, Figure S3: optimized geometries of the excited state of studied dyes, Figure S4: ^1^HNMR spectrum of compound CA-1, Figure S5: ^13^CNMR spectrum of compound CA-1, Figure S6: ^1^HNMR spectrum of compound CA-2, Figure S7: ^1^HNMR spectrum of compound Py-1.

## Sample Availability

Data is available in this article and Supplementary Materials.
