# Supplementary material for: Synthesis and Computational Characterization of Organic UV-Dyes for Cosensitization of Transparent Dye-Sensitized Solar Cells
Source: Molecules. 2021 Dec 3;26(23):7336. doi: 10.3390/molecules26237336 (PMC8658785; doi:10.3390/molecules26237336)

Supporting Information

Synthesis and Computational Characterization of Organic UV-Dyes for Cosensitization of Transparent Dye-Sensitized Solar Cells.

Rua B. Alnoman [a] , Eman Nabil [b*], Shazia Parveen [a] , Mohamed Hagar [a,b*], Mohamed Zakaria [b] and Ahmed A. Hasanein [b]

a. Faculty of Science, Chemistry Department, Taibah University, Yanbu, 46423, Saudi Arabia.
b. Faculty of Science, Chemistry Department, Alexandria University, Alexandria, 21321, Egypt.

***Corresponding authors:**
Mohamedhaggar@gmail.com (Mohamed Hagar);
eman.nabil@alexu.edu.eg (Eman Nabil)

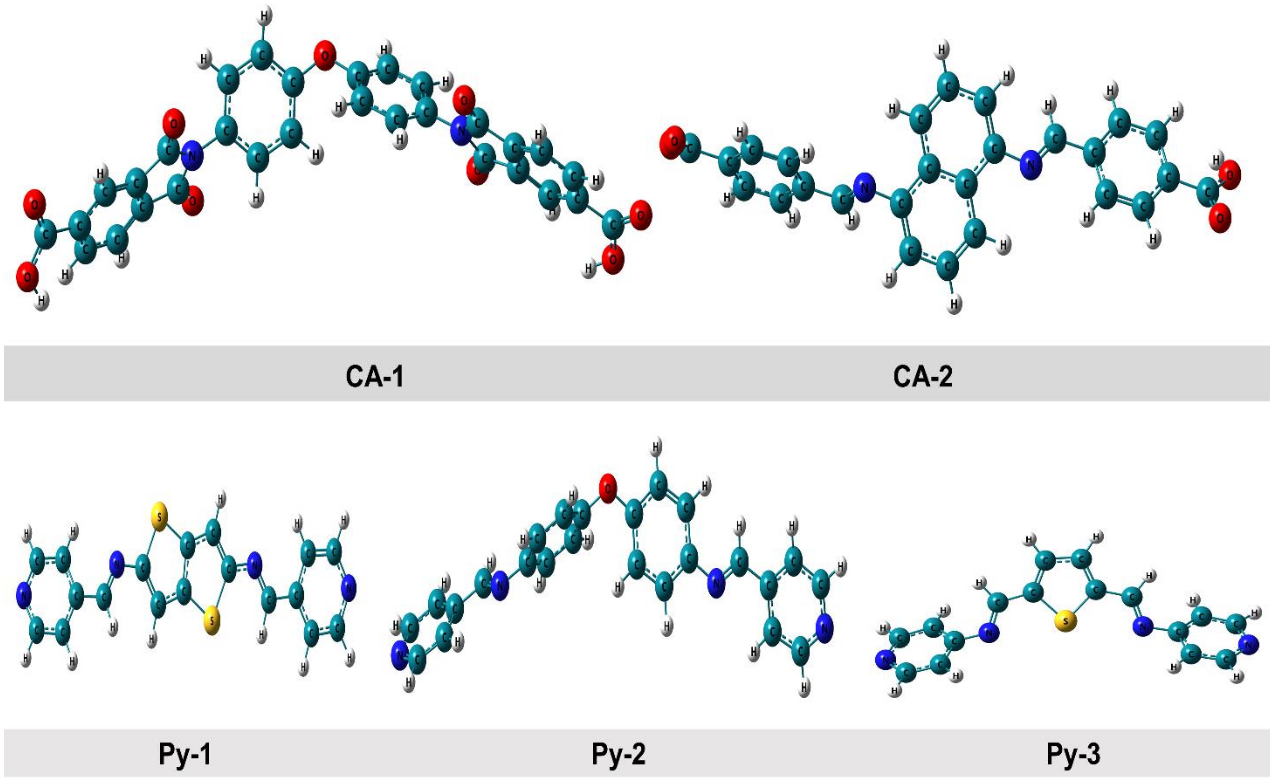

Figure S1. Optimized Geometries of the Ground State of the Studied dyes

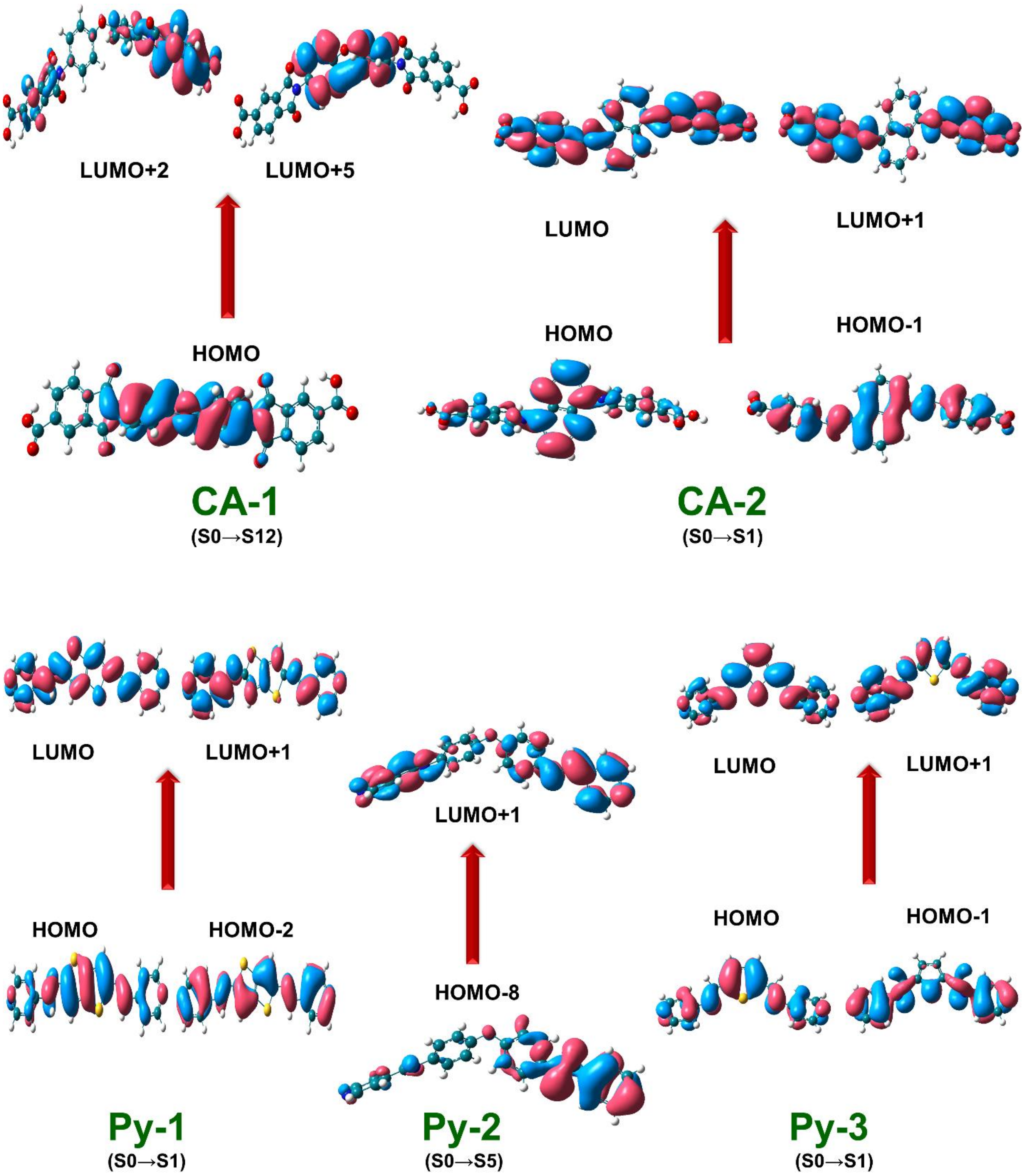

Figure S2. Major Contributing Molecular Orbitals in the Studied Electronic Transitions.

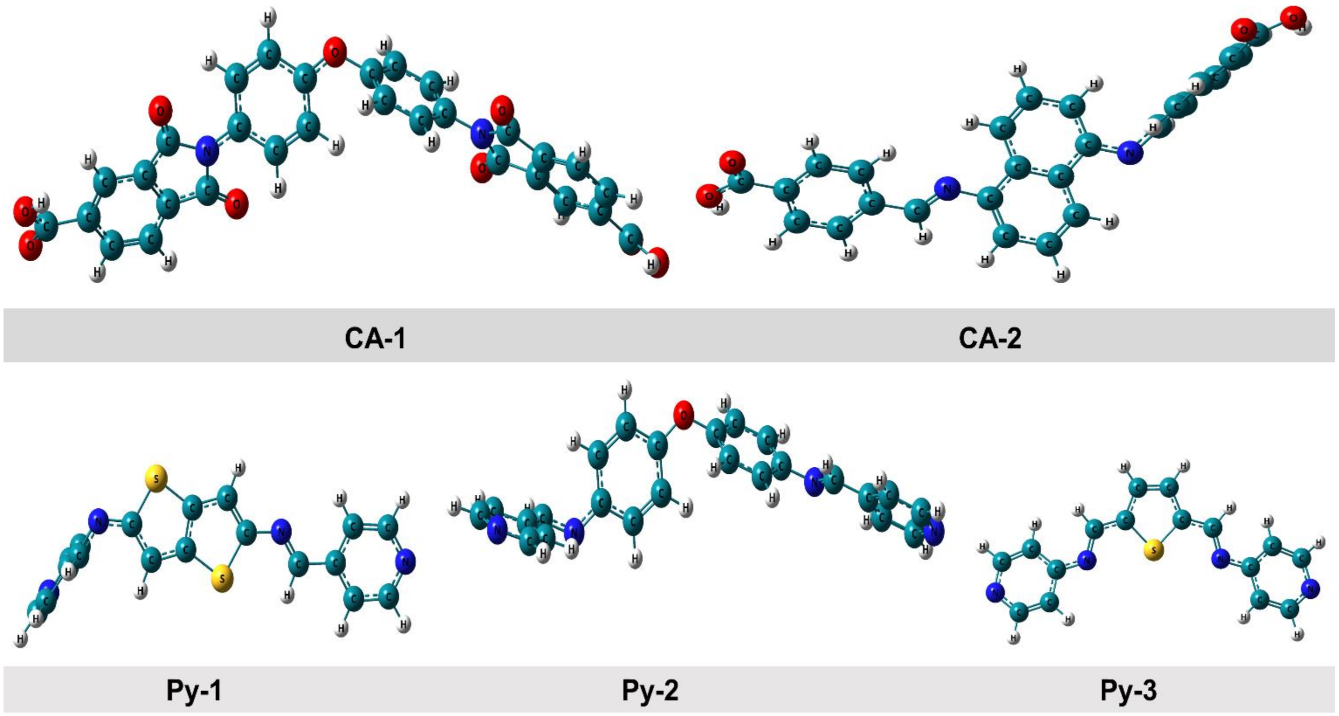

Figure S3. Optimized geometries of the Excited State of studied Dyes

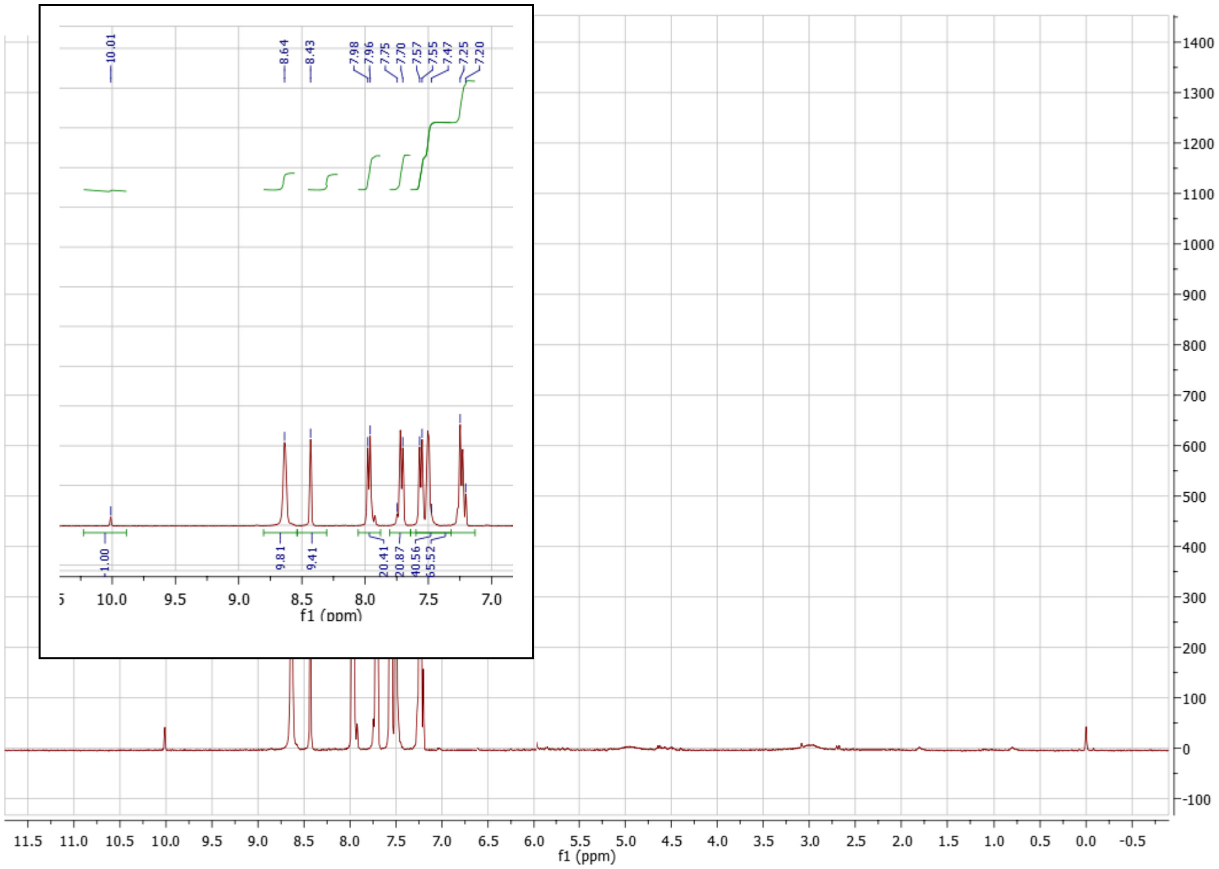

Figure S4. **¹HNMR** (500 MHz, DMSO-*d*₆) of 2,2'-[Oxybis(4,1-phenylene)]bis(1H-isoindole- 1,3(2H)-dione-5-carboxylic acid)**, CA-1**

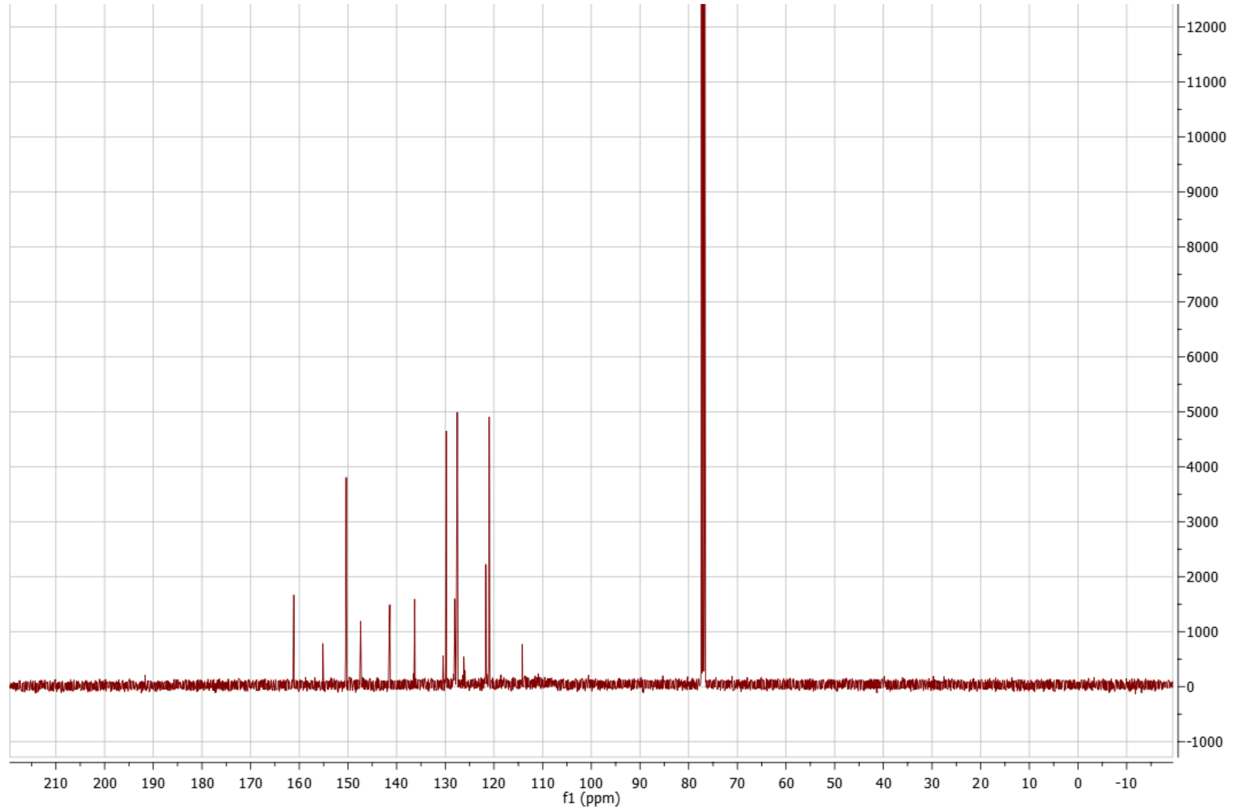

Figure S5. **^{13}CNMR** (175 MHz, $CDCl_3$) of 2,2'-[Oxybis(4,1-phenylene)]bis(1H-isoindole- 1,3(2H)-dione-5-carboxylic acid)**, CA-1.**

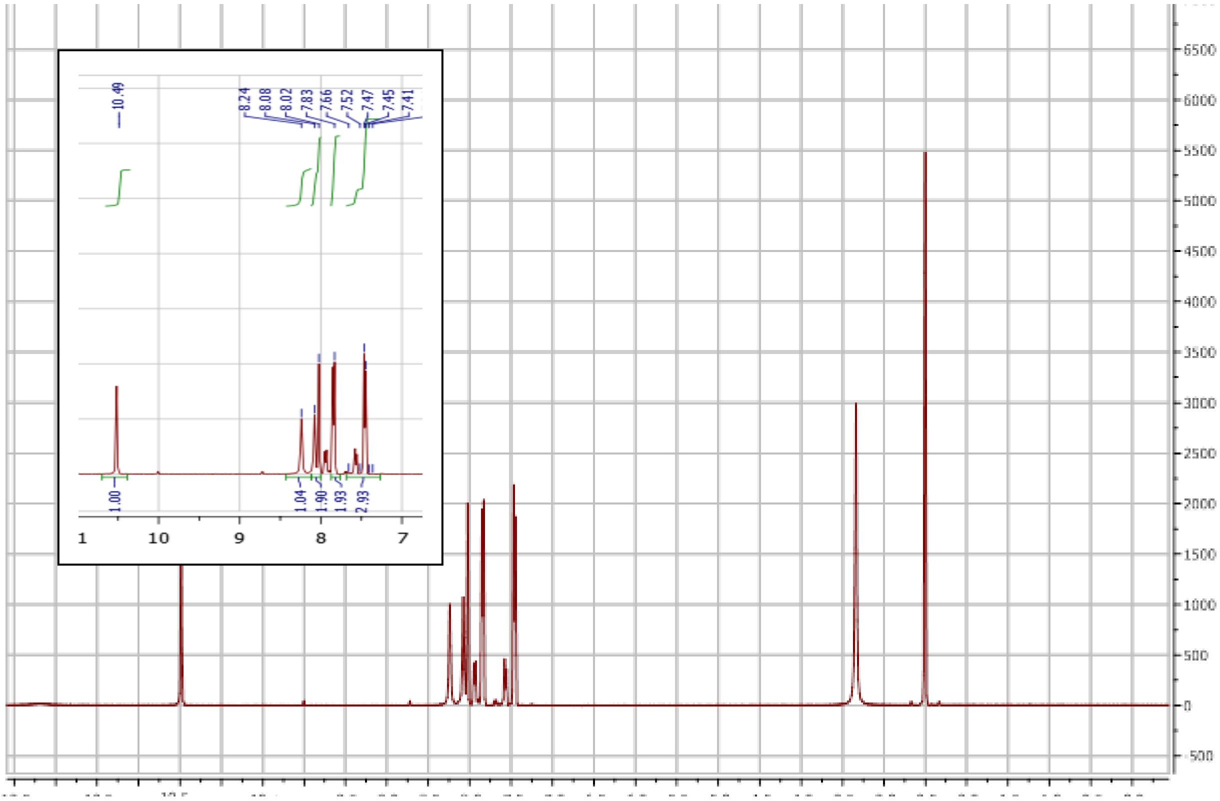

Figure S6. **^{1}HNMR** (500 MHz, DMSO-d_6) of diphenylidene-4,4'-dicarboxy-naphthalene-1,5-diamine**, CA-2**

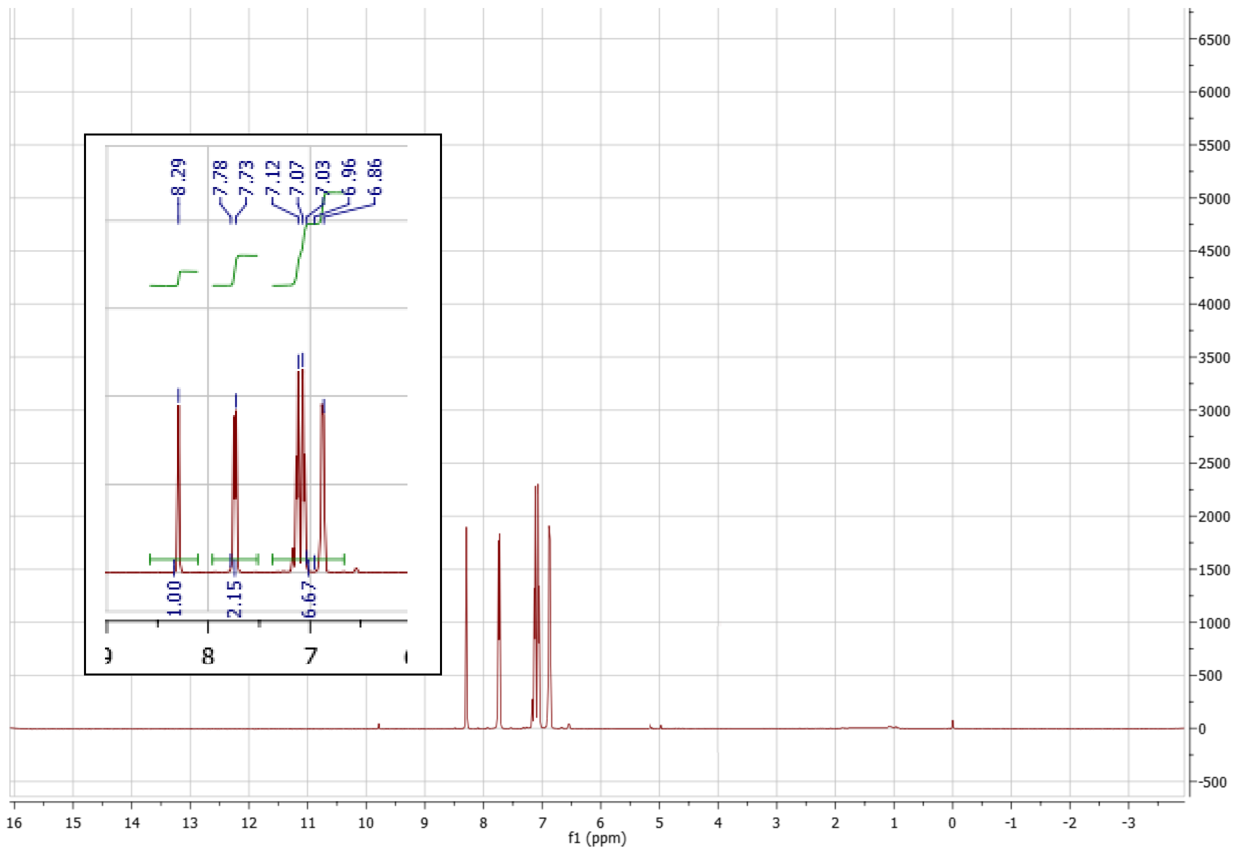

Figure S7. ^{1}**HNMR** (500 MHz, $CDCl_3$) of 4-(pyridin-4-ylimino)methyl)thieno[3,2-b]thio-phen-5-yl)methylene)pyridin-4-amine**, Py-1**

Atomic Coordinates of Isolated Dyes:

Below, we provide the atomic positions (coordinates) for the optimized geometries of the isolated dyes obtained by DFT-M06-2X/6-311G (d,p).

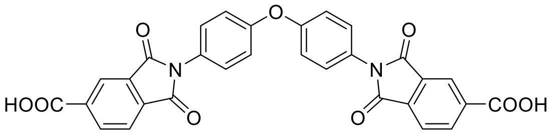

CA-1 Dye.

C	1.19045763	2.36494441	-0.17513121
O	0.00558838	3.05062043	-0.31563801
C	-1.17985066	2.35393687	-0.30377278
C	-2.27132280	2.98196428	0.28496031
C	-3.51420909	2.36614586	0.26037101
C	-3.65090924	1.11345281	-0.33106610
C	-2.55327968	0.48268493	-0.91032880
C	-1.31462180	1.10728049	-0.90915636
C	1.34643365	1.35484865	0.77012619
C	2.57931451	0.73360828	0.90551159
C	3.65149095	1.14101701	0.11535640
C	3.49460732	2.16285515	-0.81646855
C	2.25520041	2.76806439	-0.97116918
N	-4.92166995	0.47232479	-0.35016803
N	4.91922547	0.51233834	0.26633691
C	-5.50393796	-0.08120798	-1.50237914
C	-6.81190028	-0.64983746	-1.06008036
C	-6.95471543	-0.41163176	0.29888043
C	-5.74134534	0.31075481	0.78104751
C	5.69602909	0.03780064	-0.80437991
C	6.93275289	-0.52269704	-0.18672282
C	6.85230889	-0.35939713	1.18591839
C	5.55386975	0.30839823	1.50303954
C	-7.78221911	-1.30222017	-1.79243640
C	-8.91625770	-1.73274397	-1.10242485
C	-9.05667250	-1.50381189	0.26935652
C	-8.07349928	-0.81837684	0.99181308
C	8.02565303	-1.11336954	-0.78750390
C	9.05926992	-1.53751817	0.05388514
C	8.98593119	-1.34960802	1.43805598
C	7.86956823	-0.76389432	2.02953161
O	-5.48549941	0.68764900	1.89077383
O	-5.02428637	-0.08048518	-2.60211259
O	5.39775674	0.08812854	-1.96548696
O	5.11811549	0.62080938	2.57613414
C	-10.28084443	-1.92920415	1.02701304
O	-10.62270658	-1.39636358	2.04579764
O	-11.00001118	-2.93932251	0.52177255
C	10.31445914	-2.16217087	-0.48274769
O	11.35447294	-2.13737045	0.11400619
O	10.24113285	-2.75494548	-1.68185587
H	-2.13597463	3.95325650	0.74351394
H	-4.37309850	2.85033905	0.70644700
H	-2.67156495	-0.48957832	-1.37092490
H	-0.46004396	0.62827971	-1.37064644
H	0.50939229	1.05459004	1.38860266
H	2.71442155	-0.05642666	1.63252670
H	4.33460928	2.47431499	-1.42340050
H	2.10467191	3.55923225	-1.69437620
H	-7.67161540	-1.47072809	-2.85597568
H	-9.70413103	-2.22564722	-1.66055701
H	-8.20137004	-0.62772919	2.05008030
H	8.07018800	-1.20428932	-1.86724480
H	9.82193880	-1.67459699	2.04444796
H	7.80364513	-0.62635814	3.10131482
H	-10.54119826	-3.38515808	-0.19982485
H	9.33017104	-2.86230848	-1.97888468

CA-2 Dye.

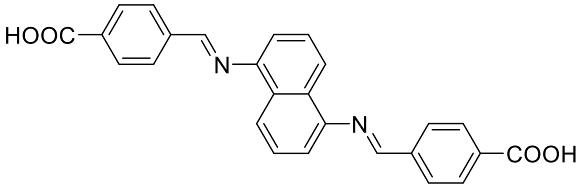

C	0.00144459	-2.52471399	0.48364073
C	-1.35465188	-2.13475022	0.46685117
C	-1.69870206	-0.80105552	0.45126373
C	-0.68004482	0.20419555	0.47150832
C	0.68004515	-0.20419689	0.47150838
C	0.99957376	-1.58644563	0.47763508
C	-0.99957350	1.58644432	0.47763648
C	-0.00144446	2.52471270	0.48364350
C	1.35465210	2.13474888	0.46685409
C	1.69870226	0.80105430	0.45126516
N	3.03851953	0.37268380	0.48511963
C	3.88289449	0.93042745	-0.28336176
C	5.31308304	0.57618278	-0.25247071
N	-3.03851913	-0.37268485	0.48511816
C	-3.88289459	-0.93042777	-0.28336326
C	-5.31308325	-0.57618226	-0.25247210
C	6.18142306	1.17800489	-1.16222378
C	7.53257614	0.85445518	-1.16142926
C	8.02226095	-0.07297683	-0.24351882
C	7.15705583	-0.66043772	0.68452016
C	5.81086934	-0.34662257	0.67528640
C	-6.18142295	-1.17800026	-1.16222828
C	-7.53257599	-0.85445027	-1.16143317
C	-8.02226100	0.07297790	-0.24351888
C	-7.15705630	0.66043485	0.68452277
C	-5.81086986	0.34661958	0.67528831
C	9.47069681	-0.44256394	-0.16774349
O	9.97592371	-0.90111695	0.82057777
O	10.21865176	-0.23811343	-1.26332476
C	-9.47069687	0.44256451	-0.16774229
O	-9.97592440	0.90111168	0.82058134
O	-10.21865150	0.23811895	-1.26332475
H	0.24668380	-3.57974071	0.50689064
H	-2.13422595	-2.88698913	0.50262215
H	2.04055140	-1.88245039	0.48841027
H	-2.04055120	1.88244885	0.48841168
H	-0.24668367	3.57973941	0.50689468
H	2.13422628	2.88698760	0.50262616
H	3.58593816	1.69183579	-1.01442918
H	-3.58593887	-1.69183614	-1.01443083
H	5.80109421	1.90567116	-1.87010349
H	8.19023948	1.35857386	-1.86015154
H	7.55957450	-1.36476442	1.40178206
H	5.12798245	-0.80094591	1.38229640
H	-5.80109363	-1.90566321	-1.87011110
H	-8.19023916	-1.35856557	-1.86015804
H	-7.55957524	1.36475869	1.40178730
H	-5.12798327	0.80093993	1.38230045
H	9.67737805	-0.02050842	-2.03094422
H	-9.67737710	0.02051986	-2.03094547

Py-1 Dye.

N	-3.67745617	0.53900108	-0.13777451
C	-4.38672325	-0.46377356	0.20413457
N	3.65247153	0.50856739	0.06169106
C	4.41317122	-0.50734309	-0.06406075
C	0.08451343	1.10089401	0.05585659
C	-0.11126436	-0.25764255	-0.14226342
C	-1.47089232	-0.64581700	-0.23262711
C	-2.29622428	0.44113912	-0.08955075
S	-1.42217288	1.94603846	0.11698408
C	1.44586610	1.48020827	0.14833608
C	2.28114565	0.40376637	0.01164129
S	1.39176506	-1.11619880	-0.23292784
H	-3.94557324	-1.38747876	0.59195522
H	4.02697500	-1.52092430	-0.21832956
H	-1.82405965	-1.64950771	-0.42208135
H	1.82220259	2.48129670	0.30160285
C	-5.85410010	-0.42763558	0.10845515
C	-6.60187608	-1.53842687	0.49209677
C	-6.53611702	0.69781749	-0.35734957
C	-7.98809150	-1.47679353	0.39345271
H	-6.11959092	-2.43626660	0.86037712
C	-7.92044587	0.65610235	-0.41274321
H	-5.99004335	1.58018935	-0.66454830
N	-8.64794288	-0.40645594	-0.04827168
H	-8.59203705	-2.33029627	0.68428289
H	-8.47685262	1.51763744	-0.76758036
C	5.87560549	-0.37280546	-0.00878436
C	6.68553556	-1.49778584	-0.14820066
C	6.49556405	0.86409113	0.18068835
C	8.06605867	-1.33885550	-0.09347833
H	6.25407507	-2.48093700	-0.29611220
C	7.88005578	0.91209217	0.21972682
H	5.90167139	1.76171810	0.29309178
N	8.66660135	-0.16228297	0.08630807
H	8.71697516	-2.20089661	-0.19891634
H	8.38637855	1.86083259	0.36570708

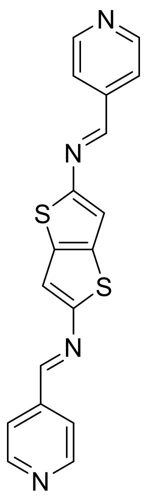

Py-2 Dye.

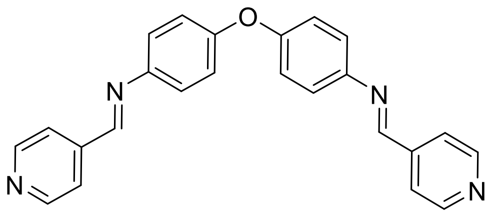

N	4.80575100	-0.47452339	-0.13623221
C	5.90243557	0.13424147	0.06339972
N	-4.98273902	0.08667603	0.60073746
C	-5.77197380	-0.08465884	-0.37915134
C	-1.94423705	2.10907033	1.10882302
C	-3.19418175	1.53476375	1.29428328
C	-3.70373418	0.63211972	0.35874507
C	-2.92355504	0.27482402	-0.74714910
C	-1.66848139	0.83821976	-0.93023252
C	-1.19148386	1.75957813	-0.00459862
C	3.51052661	1.54574849	-0.73690353
C	2.29875620	2.21687686	-0.74904898
C	1.17030911	1.62463458	-0.18673603
C	1.24428323	0.34852130	0.36670139
C	2.45775551	-0.32646028	0.35218669
C	3.60609585	0.26850761	-0.17120904
O	0.02604685	2.38328106	-0.19367372
H	5.95109025	1.21208706	0.25539306
H	-5.51340139	0.21584657	-1.40095940
H	-1.54189426	2.82273820	1.81708297
H	-3.79535590	1.78649785	2.15942283
H	-3.29003377	-0.46191703	-1.45223972
H	-1.05608450	0.56385962	-1.78142188
H	4.37833033	2.00078647	-1.19969003
H	2.20498104	3.19919879	-1.19531098
H	0.36729152	-0.11554257	0.79938603
H	2.53348539	-1.32326809	0.76961691
C	-7.10745298	-0.68543912	-0.19866638
C	-7.95809530	-0.82430221	-1.29157944
C	-7.55683411	-1.12503318	1.04701860
C	-9.21309015	-1.39168453	-1.09408220
H	-7.65432930	-0.49848093	-2.27948652
C	-8.82572743	-1.67685035	1.13540708
H	-6.92291770	-1.03501864	1.91954875
N	-9.65122913	-1.81433134	0.09157052
H	-9.89495372	-1.50994681	-1.93003347
H	-9.20093003	-2.02825374	2.09123878
C	7.19277064	-0.58098256	0.07207002
C	7.28104656	-1.95233666	-0.16970149
C	8.36941255	0.11723492	0.32969619
C	8.53118849	-2.55082814	-0.14054815
H	6.38835745	-2.52842686	-0.37579462
C	9.57350278	-0.57921471	0.33539094
H	8.35386050	1.18351995	0.52273399
N	9.66765939	-1.88882932	0.10628581
H	8.62896452	-3.61586083	-0.32506781
H	10.50365276	-0.05631352	0.53374886

Py-3 Dye.

C	2.63829573	0.95727811	0.23562846
N	3.02292338	-0.23629418	0.01139100
C	-2.63829488	0.95726448	0.23578660
N	-3.02292444	-0.23628532	0.01143141
C	1.22892219	1.29918044	0.30964505
C	0.70701550	2.54314823	0.56562541
C	-0.70699665	2.54314745	0.56567388
C	-1.22891951	1.29918347	0.30971350
S	-0.00000582	0.10687084	0.06634720
H	3.33808039	1.78398505	0.39837200
H	-3.33808085	1.78395196	0.39862298
H	1.32236562	3.41457416	0.74604022
H	-1.32233442	3.41457343	0.74613054
C	4.40019282	-0.48887597	-0.09593918
C	4.92750955	-1.62740362	0.51466074
C	5.26761579	0.32210657	-0.83050150
C	6.28639113	-1.87712026	0.39450123
H	4.28331296	-2.28977682	1.07849331
C	6.60974696	-0.03079331	-0.89522611
H	4.90338614	1.19269472	-1.36195526
N	7.12990544	-1.10325228	-0.29593292
H	6.71921738	-2.74943624	0.87400108
H	7.29916336	0.58453045	-1.46506829
C	-4.40019571	-0.48887138	-0.09592011
C	-4.92754904	-1.62733312	0.51476355
C	-5.26758005	0.32204435	-0.83061127
C	-6.28643209	-1.87703953	0.39457145
H	-4.28339374	-2.28966270	1.07869378
C	-6.60971034	-0.03084296	-0.89536050
H	-4.90329770	1.19256501	-1.36213912
N	-7.12990575	-1.10323757	-0.29598127
H	-6.71928924	-2.74930473	0.87413536
H	-7.29909526	0.58442000	-1.46530659

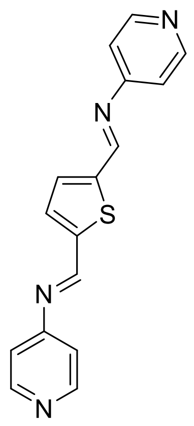

Supplement: Supplementary file 1 [file molecules-26-07336-s001.zip › SI_20th Nov.pdf]
